# Psoriasis Pathogenesis and Treatment

**DOI:** 10.3390/ijms20061475

**Published:** 2019-03-23

**Authors:** Adriana Rendon, Knut Schäkel

**Affiliations:** Department of Dermatology, Heidelberg University, 69120 Heidelberg, Germany; RuthAdriana.RendonMedina@med.uni-heidelberg.de

**Keywords:** psoriasis, inflammation, chronic skin disease

## Abstract

Research on psoriasis pathogenesis has largely increased knowledge on skin biology in general. In the past 15 years, breakthroughs in the understanding of the pathogenesis of psoriasis have been translated into targeted and highly effective therapies providing fundamental insights into the pathogenesis of chronic inflammatory diseases with a dominant IL-23/Th17 axis. This review discusses the mechanisms involved in the initiation and development of the disease, as well as the therapeutic options that have arisen from the dissection of the inflammatory psoriatic pathways. Our discussion begins by addressing the inflammatory pathways and key cell types initiating and perpetuating psoriatic inflammation. Next, we describe the role of genetics, associated epigenetic mechanisms, and the interaction of the skin flora in the pathophysiology of psoriasis. Finally, we include a comprehensive review of well-established widely available therapies and novel targeted drugs.

## 1. Definition and Epidemiology 

Psoriasis is a chronic inflammatory skin disease with a strong genetic predisposition and autoimmune pathogenic traits. The worldwide prevalence is about 2%, but varies according to regions [1]. It shows a lower prevalence in Asian and some African populations, and up to 11% in Caucasian and Scandinavian populations [2,3,4,5]. 

### 1.1. Clinical Classification

**Figure 1:**
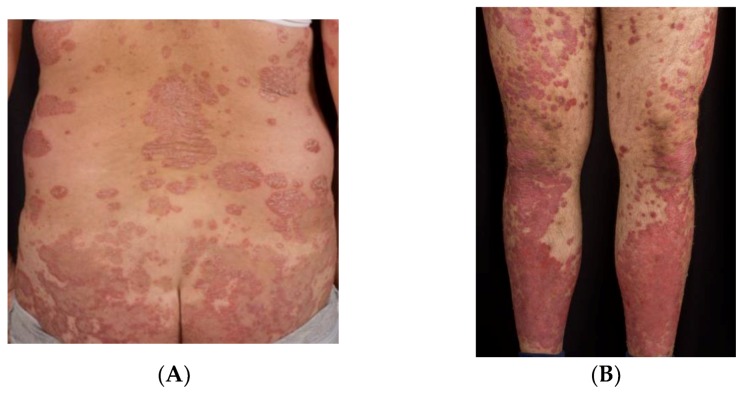

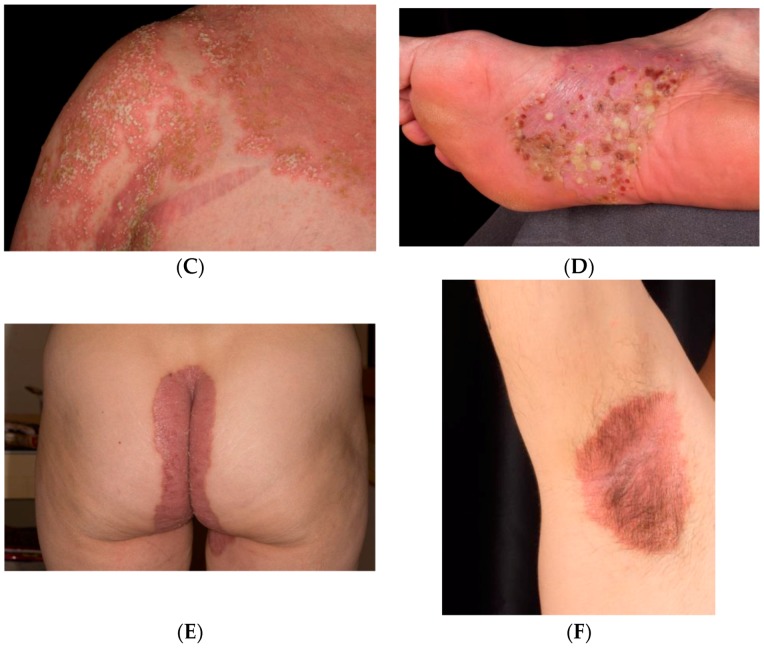
Clinical manifestations of psoriasis. (**A**,**B**) Psoriasis vulgaris presents with erythematous scaly plaques on the trunk and extensor surfaces of the limbs. (**C**) Generalized pustular psoriasis. (**D**) Pustular psoriasis localized to the soles of the feet. This variant typically affects the palms of the hands as well; hence, psoriasis pustulosa palmoplantaris. (**E**,**F**) Inverse psoriasis affects the folds of the skin (i.e., axillary, intergluteal, inframammary, and genital involvement).

The dermatologic manifestations of psoriasis are varied; psoriasis vulgaris is also called plaque-type psoriasis, and is the most prevalent type. The terms psoriasis and psoriasis vulgaris are used interchangeably in the scientific literature; nonetheless, there are important distinctions among the different clinical subtypes (See Figure 1). 

### 1.2. Psoriasis Vulgaris

About 90% of psoriasis cases correspond to chronic plaque-type psoriasis. The classical clinical manifestations are sharply demarcated, erythematous, pruritic plaques covered in silvery scales. The plaques can coalesce and cover large areas of skin. Common locations include the trunk, the extensor surfaces of the limbs, and the scalp [6,7]. 

### 1.3. Inverse Psoriasis 

Also called flexural psoriasis, inverse psoriasis affects intertriginous locations, and is characterized clinically by slightly erosive erythematous plaques and patches. 

### 1.4. Guttate Psoriasis

Guttate psoriasis is a variant with an acute onset of small erythematous plaques. It usually affects children or adolescents, and is often triggered by group-A streptococcal infections of tonsils. About one-third of patients with guttate psoriasis will develop plaque psoriasis throughout their adult life [8,9].

### 1.5. Pustular psoriasis

Pustular psoriasis is characterized by multiple, coalescing sterile pustules. Pustular psoriasis can be localized or generalized. Two distinct localized phenotypes have been described: psoriasis pustulosa palmoplantaris (PPP) and acrodermatitis continua of Hallopeau. Both of them affect the hands and feet; PPP is restricted to the palms and soles, and ACS is more distally located at the tips of fingers and toes, and affects the nail apparatus. Generalized pustular psoriasis presents with an acute and rapidly progressive course characterized by diffuse redness and subcorneal pustules, and is often accompanied by systemic symptoms [10]. 

**Figure 2:**
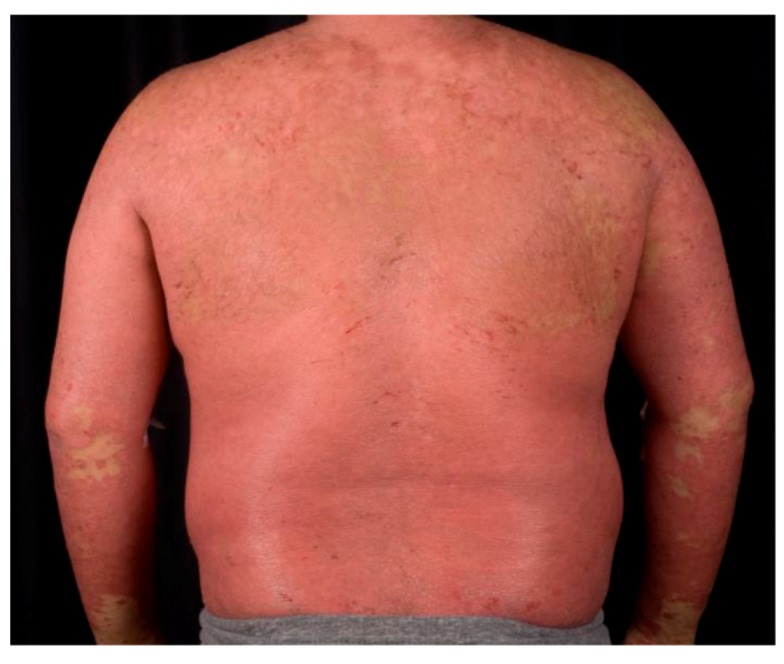
Erythrodermic psoriasis.

*Erythrodermic psoriasis* is an acute condition in which over 90% of the total body surface is erythematous and inflamed. Erythroderma can develop on any kind of psoriasis type, and requires emergency treatment (Figure 2). 

### 1.6. Comorbidities in Psoriasis

Psoriasis typically affects the skin, but may also affect the joints, and has been associated with a number of diseases. Inflammation is not limited to the psoriatic skin, and has been shown to affect different organ systems. Thus, it has been postulated that psoriasis is a systemic entity rather than a solely dermatological disease. When compared to control subjects, psoriasis patients exhibit increased hyperlipidemia, hypertension, coronary artery disease, type 2 diabetes, and increased body mass index. The metabolic syndrome, which comprises the aforementioned conditions in a single patient, was two times more frequent in psoriasis patients [11,12]. Coronary plaques are also twice as common in psoriasis patients when compared to control subjects [13]. Several large studies have shown a higher prevalence of diabetes and cardiovascular disease correlating with the severity of psoriasis [14,15,16,17,18]. There are divided opinions regarding the contribution of psoriasis as an independent cardiovascular risk factor [19,20]; however, the collective evidence supports that psoriasis independently increases risk for myocardial infarction, stroke, and death due to cardiovascular disease (CVD) [21,22,23,24,25,26,27,28]. In addition, the risk was found to apply also to patients with mild psoriasis to a lower extent [21,27]. 

Vascular inflammation assessed via 18F-fluorodeoxyglucose positron emission tomography-computed tomography (18F-FDG PET/CT) found psoriasis duration to be a negative predicting factor. It was suggested that the cumulative effects of low-grade chronic inflammation might accelerate vascular disease development [29]. In a study by Metha et al., systemic and vascular inflammation in six patients with moderate to severe psoriasis was quantified by FDG-PET/CT. Inflammation foci were registered as expected in the skin, joints, and tendons. In addition, FDG uptake in the liver and aorta revealed subclinical systemic inflammation [30]. Furthermore, standardized uptake values were reduced in the liver, spleen, and aorta following treatment with ustekinumab {Kim, 2018 #359}. A new biomarker to assess CVD risk in psoriasis patients was proposed by nuclear magnetic resonance spectroscopy [31]. The signal originating from glycan N-acetylglucosamine residues called GlycA in psoriasis patients was associated with psoriasis severity and subclinical CVD, and was shown to be reduced in response to the effective treatment of psoriasis. 

**Figure 3:**
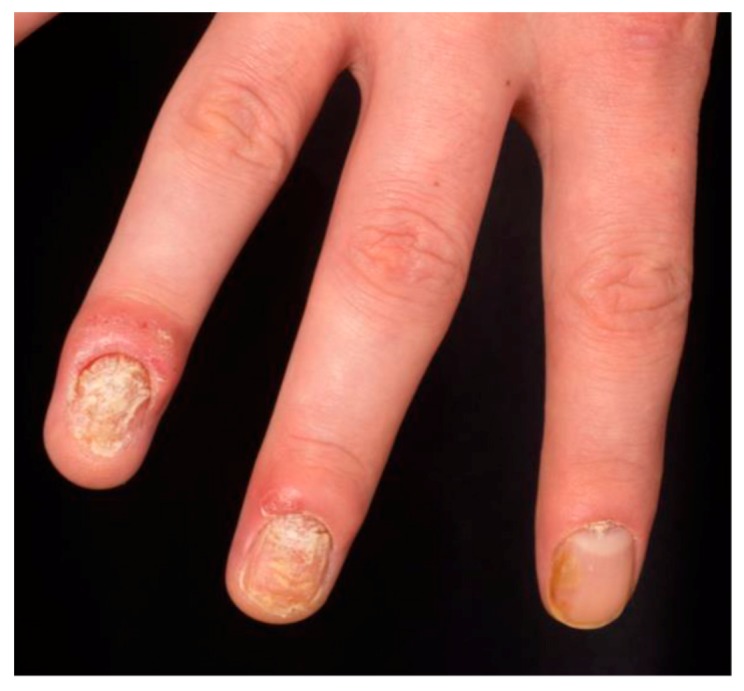
Onycholysis and oil drop changes on psoriatic nail involvement.

Psoriatic inflammation of the joints results in psoriatic arthritis (PsA). The skin manifestations generally precede PsA, which shares the inflammatory chronicity of psoriasis and requires systemic therapies due to a potential destructive progression. Psoriatic arthritis develops in up to 40% of psoriasis patients [32,33,34,35,36,37,38]; around 15% of psoriasis patients are thought to have undiagnosed PsA [39]. It presents clinically with dactylitis and enthesitis in oligoarticular or polyarticular patterns. The polyarticular variant is frequently associated with nail involvement [40]. Nails are specialized dermal appendages that can also be affected by psoriatic inflammation. Nail psoriasis is reported to affect more than half of psoriasis patients, and can present as the only psoriasis manifestation in 5–10% of patients [41]. The clinical presentation of nail psoriasis depends on the structure affected by the inflammatory process. Nail matrix involvement presents as pitting, leukonychia, and onychodystrophy, whereas inflammation of the nail bed presents as oil-drop discoloration, splinter hemorrhages, and onycholysis (Figure 3) [42]. Psoriatic nail involvement is associated with joint involvement, and up to 80% of patients with PsA have nail manifestations [43,44]. 

In addition to an increased risk for cardiometabolic disease, psoriasis has been associated with a higher prevalence of gastrointestinal and chronic kidney disease. Susceptibility loci shared between psoriasis and inflammatory bowel disease support this association in particular with regard to Crohn’s disease [45,46]. An association with mild liver disease, which correlates with imaging studies, has been reported [30,47]. Psoriasis might be a risk factor for chronic kidney disease and end-stage renal disease, independent of traditional risk factors (demographic, cardiovascular, or drug-related) [48]. 

Taken together, the different factors contributing to psoriasis as a systemic disease can have a dramatic effect on the quality of life of patients and their burden of disease. Psoriasis impairment to psychological quality of life is comparable to cancer, myocardial infarction, and depression [49]. The high burden of disease is thought to be owed to the symptoms of the disease, which include pain, pruritus, and bleeding, in addition to the aforementioned associated diseases [50]. The impact of psoriasis on psychological and mental health is currently an important consideration due to the implications of the disease on social well-being and treatment. Patients with psoriasis have an increased prevalence of depression and anxiety and suicidal ideation. Interestingly, psoriasis treatment leads to improvement in anxiety symptoms [51,52]. 

## 2. Pathogenesis

**Figure 4:**
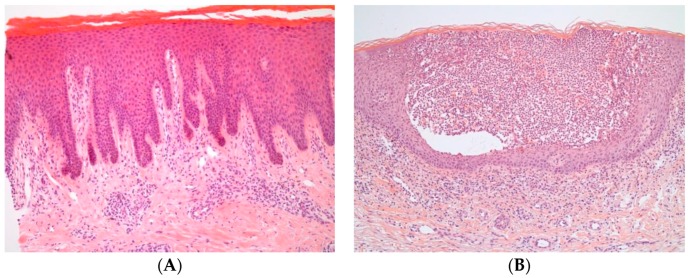
Histopathology of psoriasis. (**A**) Psoriasis vulgaris characteristically shows acanthosis, parakeratosis, and dermal inflammatory infiltrates. (**B**) In pustular psoriasis, acanthotic changes are accompanied by epidermal predominantly neutrophilic infiltrates, which cause pustule formation.

The hallmark of psoriasis is sustained inflammation that leads to uncontrolled keratinocyte proliferation and dysfunctional differentiation. The histology of the psoriatic plaque shows acanthosis (epidermal hyperplasia), which overlies inflammatory infiltrates composed of dermal dendritic cells, macrophages, T cells, and neutrophils (Figure 4). Neovascularization is also a prominent feature. The inflammatory pathways active in plaque psoriasis and the rest of the clinical variants overlap, but also display discrete differences that account for the different phenotype and treatment outcomes.

### 2.1. Main Cytokines and Cell Types in Plaque Psoriasis

Disturbances in the innate and adaptive cutaneous immune responses are responsible for the development and sustainment of psoriatic inflammation [53,54]. An activation of the innate immune system driven by endogenous danger signals and cytokines characteristically coexists with an autoinflammatory perpetuation in some patients, and T cell-driven autoimmune reactions in others. Thus, psoriasis shows traits of an autoimmune disease on an (auto)inflammatory background [55], with both mechanisms overlapping and even potentiating one another. 

**Figure 5:**
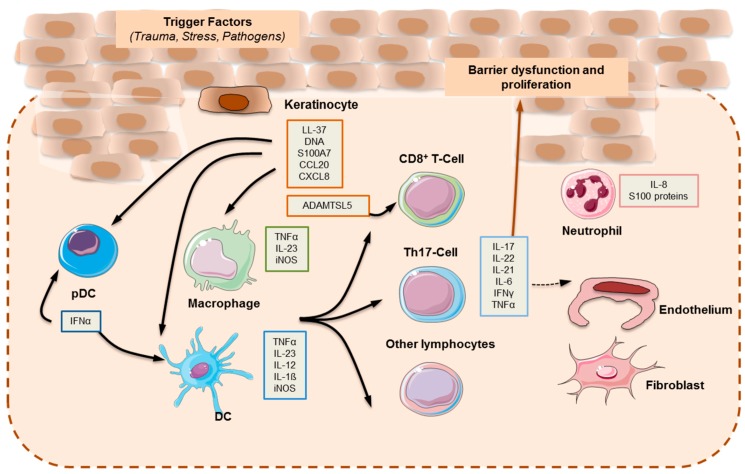
The pathogenesis of psoriasis.

The main clinical findings in psoriasis are evident at the outermost layer of the skin, which is made up of keratinocytes. However, the development of the psoriatic plaque is not restricted to inflammation in the epidermal layer, but rather is shaped by the interaction of keratinocytes with many different cell types (innate and adaptive immune cells, vasculature) spanning the dermal layer of the skin. The pathogenesis of psoriasis can be conceptualized into an initiation phase possibly triggered by trauma (Koebner phenomenon), infection, or drugs [53] and a maintenance phase characterized by a chronic clinical progression (see Figure 5). 

It is well known that dendritic cells play a major role in the initial stages of disease. Dendritic cells are professional antigen-presenting cells. However, their activation in psoriasis is not entirely clear. One of the proposed mechanisms involves the recognition of antimicrobial peptides (AMPs), which are secreted by keratinocytes in response to injury and are characteristically overexpressed in psoriatic skin. Among the most studied psoriasis-associated AMPs are LL37, β-defensins, and S100 proteins [56]. LL37 or cathelicidin has been attributed a pathogenic role in psoriasis. It is released by damaged keratinocytes, and subsequently forms complexes with self-genetic material from other damaged cells. LL37 bound to DNA stimulates toll-like receptor (TLR) 9 in plasmacytoid dendritic cells (pDCs) [57]. The activation of pDC is key in starting the development of the psoriatic plaque, and is characterized by the production of type I IFN (IFN-α and IFN-β). Type I IFN signaling promotes myeloid dendritic cells (mDC) phenotypic maturation, and has been implicated in Th1 and Th17 differentiation and function, including IFN-γ and interleukin (IL)-17 production, respectively [58,59,60]. 

Whilst LL37–DNA complexes stimulate pDCs through TLR9, LL37 bound to RNA stimulates pDCs through TLR7. In addition, LL37–RNA complexes act on mDCs via TLR8 [56,57]. Activated mDCs migrate into draining lymph nodes and secrete tumor necrosis factor (TNF)-α, IL-23, and IL-12, with the latter two modulating the differentiation and proliferation of Th17 and Th1 cell subsets, respectively. Furthermore, slan^+^ monocytes, which are important pro-inflammatory cells found in psoriasis skin lesions, respond to LL37–RNA activation by secreting high amounts of TNF-α, IL-12, and IL-23 [61].

The activation of the adaptive immune response via the distinct T cell subsets drives the maintenance phase of psoriatic inflammation [62]. Th17 cytokines, namely IL-17, IL-21, and IL-22 activate keratinocyte proliferation in the epidermis. 

The inflammatory milieu activates keratinocyte proliferation via TNF-α, IL-17, and IFN-γ. Keratinocytes are also activated by LL37 and DNA, and greatly increase the production of type I IFNs [57]. Furthermore, they participate actively in the inflammatory cascade through cytokine (IL-1, IL-6, and TNF-α), chemokine, and AMP secretion.

A widely used psoriasis-like inflammation mouse model relies on the effect of the TLR7/8 agonist imiquimod, and is thus in support of the TLR7/8 disease initiation model. In addition, the response to imiquimod was blocked in mice deficient of IL-23 or IL-17R, which highlights the involvement of the IL-23/IL-17 axis in skin inflammation and psoriasis-like pathology [63]. 

The TNFα–IL-23–Th17 inflammatory pathway characterizes plaque-type psoriasis. The IL-17 cytokine family is composed of six members: IL-17A–F. They are produced by different cell types, and are important regulators of inflammatory responses [64]. So far, the clinically relevant signaling in psoriasis is mediated mostly by IL-17A and IL-17F; both act through the same receptor, but have different potencies. IL-17A exerts a stronger effect than IL-17F, and the IL-17A/IL-17F heterodimer has an intermediate effect. IL-17A binds to its trimeric receptor complex composed of two IL-17RA subunits and one IL-17RC subunit, resulting in the recruitment of the ACT1 adaptor protein. The interaction between ACT1 and the IL-17 receptor complex leads to the activation of a series of intracellular kinases including: extracellular signal-regulated kinase (ERK), p38 MAPK, TGF-beta-activated Kinase 1 (TAK1), I-kappa B kinase (IKK), and glycogen synthase kinase 3 beta (GSK-3 beta). These kinases enable NFκB, AP-1, and C/EBP transcription of pro-inflammatory cytokines, chemokines, and antimicrobial peptides. Th1 and Th2 cytokines act through Janus kinase (JAK)-STAT signaling pathways, whereas Th17 responses are mediated by ACT1 and NFκB [65]. Alternatively, γδ T cells are able to produce IL-17A independently of the IL-23 stimulus [66]. 

Drugs targeting TNFα, IL-23, and IL-17 and signaling pathways such as JAK/STAT are effective in the clinical management of plaque psoriasis. However, alternate inflammatory pathways may be valid for distinct psoriatic variants. 

### 2.2. Pathophysiology in Variants

Whereas the TNFα–IL23–Th17 axis plays a central role in T cell-mediated plaque psoriasis, the innate immune system appears to play a more prominent role in the pustular variants of psoriasis [55]. Different pathomechanisms are associated with distinct psoriasis subtypes. 

In guttate psoriasis, streptococcal superantigens are thought to stimulate the expansion of T cells in the skin [67]. It was shown that there is a considerable sequence homology between streptococcal M proteins and human keratin 17 proteins. Molecular mimicry may play a role in patients with the major histocompatibility HLA-Cw6 allele, since CD8(+) T cell IFN-γ responses were elicited by K17 and M6 peptides in said patients [68,69].

Pustular psoriasis is characterized by the increased expression of IL-1β, IL-36α, and IL-36γ transcripts, which have been found in pustular psoriasis compared to psoriasis vulgaris [70]. Nevertheless, IL-17 signaling is also involved in pustular psoriasis and patients with generalized pustular psoriasis without IL-36R mutations responded to anti-IL-17 treatments [71,72]. 

In nail psoriasis and psoriatic arthritis (PsA), an increased expression of TNF-α, NFκB, IL-6, and IL-8 in psoriasis-affected nails is consistent with the inflammatory markers found on lesional psoriatic skin [73]. The pathophysiology of PsA and psoriasis is shared as synovial tissue in psoriatic arthritis expresses pro-inflammatory cytokines: IL-1, IFN-γ, and TNFα [74,75]. Infiltrating cells in psoriasis arthritis, tissues, and synovial fluid revealed large clonal expansions of CD8^+^ T cells. Joint pathology, specifically bone destruction, is partly mediated via IL-17A signaling, which induces the receptor activator of nuclear factor kappa b ligand (RANKL), and in turn activating osteoclasts. Pro-inflammatory cytokines IL-1β and TNF-α act in synergy with the local milleu [76]. 

### 2.3. Autoimmunity in Psoriasis

Psoriasis shows clear autoimmune-related pathomechanisms. This very important area of research will allow for a deeper understanding of to which extent autoantigen-specific T cells contribute to the development, chronification, and overall course of the disease. 

LL37 is one of two well-studied T cell autoantigens in psoriasis. CD4^+^ and CD8^+^ T cells specific for LL37 were found in two-thirds of patients with moderate to severe plaque psoriasis in a study. LL37-specific T cells produce IFN-γ, and CD4^+^ T cells produce IL-17, IL-21, and IL-22 as well. LL37-specific T cells can be found in lesional skin or in the blood, where they correlate with disease activity [77]. CD8^+^ T cells activated through LL37 engage in epidermotropism, autoantigen recognition, and the further secretion of Th17 cytokines. The melanocytic protein ADAMTSL5 was found to be an HLA-C*06:02-restricted autoantigen recognized by an autoreactive CD8^+^ T cell TCR. This finding establishes melanocytes as autoimmune target cells, but does not exclude other cellular targets [78].

Other autoantigen candidates include lipid antigens generated by phospholipase A2 (PLA2) group IVD (PLA2G4D) and hair follicle-derived keratin 17 [79,80]. Interestingly, keratin 17 exposure only lead to CD8+ T cell proliferation in patients with the HLA-Cw*0602 allele (see above) [81]. 

### 2.4. Genetics

Psoriasis has a genetic component that is supported by patterns of familial aggregation. First and second-degree relatives of psoriasis patients have an increased incidence of developing psoriasis, while monozygotic twins have a two to threefold increased risk compared to dizygotic twins [82,83]. Determining the precise effect of genetics in shaping innate and adaptive immune responses has proven problematic for psoriasis and other numerous immune-mediated diseases [84,85]. The genetic variants associated with psoriasis are involved in different biological processes, including immune functions such as antigen presentation, inflammation, and keratinocyte biology [55]. 

#### 2.4.1. Antigen Presentation

Genome-wide linkage studies of psoriasis-affected families have so far detected at least 60 chromosomal loci linked to psoriatic susceptibility [86,87,88]; the most prominent locus is PSORS1, which has been attributed up to 50% of the heritability of the disease [89]. PSORS1 is located on chromosome 6p21 within the major histocompatibility complex (MHC), which is specifically in the class I telomeric region of HLA-B, and spans an approximately 220 kb-long segment and corresponds to HLA-Cw6 (C*06:02). HLA-Cw6 is strongly linked to early and acute onset psoriasis [90,91]. The HLA-C*06:02 allele is present in more than 60% of patients, and increases the risk for psoriasis nine to 23-fold [92]. Nevertheless, no link between late-onset psoriasis or pustular psoriasis and PSORS1 could be established, possibly reflecting a genetically heterogenic background associated with different clinical phenotypes [93]. PSORS2 spans the CARD14 gene, while PSORS4 is located in the epidermal differentiation complex [94,95,96,97,98,99,100,101].

The results of numerous genome-wide association studies (GWAS) in psoriasis are consistent with the prominent role of PSORS1 as a risk factor, but have also revealed over 50 single-nucleotide polymorphisms (SNPs) to be associated to psoriasis [102,103,104]. Variants involving the adaptive and immune system are a constant result in these studies [53,103,105].

#### 2.4.2. Genetic Variants Implicated in Aberrant Keratinocyte Proliferation and Differentiation

The immunogenetics of IL-23 are strongly associated with psoriasis. IL-23 is a dimer composed of a specific subunit, p19, and a p40 subunit, which is shared with IL-12. IL-23 signals through a heterodimeric receptor expressed by both innate and adaptive immune cells, which include Th17, natural killer T, γδ T cells, and RORγt^+^ innate lymphoid cells. The IL-23R signals through JAK2/TYK2 and STAT3 [106]. SNPs in the regions coding for the IL-23 cytokine (both the p40 and p19 subunit) as well as the IL-23R have been identified to convey psoriasis risk [107,108,109]. Furthermore, these variants have been found to be associated with Crohn’s disease, psoriatic arthritis, and ankylosing spondylitis [110] [74,75]. IL-23 drives the expansion of Th17 T cells that produce IL-17A/F, which is another set of cytokines whose role is pivotal in the pathogenesis of psoriasis. Monoclonal antibodies targeting both the common p40 and the specific p19 subunit of IL-23 have proven to have high clinical efficacy [109].

As mentioned above, STAT3 is found in downstream signaling by IL-23, and is therefore essential in T cell development and Th17 polarization. STAT3 has also been detected in psoriasis GWAS, and its variants are associated with psoriasis risk [107,111]. Furthermore, transcription factor Runx1 induces Th17 differentiation by interacting with RORγt. Interestingly, the interaction of Runx1 with Foxp3 results in reduced IL-17 expression [112].

*CARD14* mapping was shown to correspond to PSORS2. The CARD family encompasses scaffolding proteins that activate NF-kB. It was suggested that in psoriasis patients with respective CARD14 mutations, a triggering event can result in an aberrant NF-kB over activation [96]. CARD14 is expressed in keratinocytes and in psoriatic skin; it is upregulated in the suprabasal epidermal layers and downregulated in the basal layers. In healthy skin, CARD14 is mainly localized in the basal layer. Mutations in *CARD14* have been shown to be associated with psoriasis, as well as with familial pityriasis rubra pilaris (PRP) [113].

The NF-kB signaling pathway is involved in the production of both IL-17 and TNF-α, and thus participates in adaptive and innate immune responses [73]; it is upregulated in psoriatic lesions and is responsive to treatment [114]. Gene variations in *NFKBIA, TNIP1*, and *TRAF3PI2* affecting NF-kB regulatory proteins have been linked to psoriasis via GWAS [102,115,116,117]. TRAF3PI2 codes for the ACT1 adaptor protein and the specific variant TRAF3IP2 p. Asp10Asn was associated to both psoriasis and psoriatic arthritis [117].

The different clinical psoriasis variants may have additional genetic modifiers. For instance, mutations in the antagonist to the IL-36 receptor (IL-36RN), belonging to the IL-1 pro-inflammatory cytokine family, have been linked to pustular psoriasis [118,119]. Recessive mutations in *IL36RN*, coding for the IL-36 receptor antagonist, have been associated with generalized pustular psoriasis (GPP). This mutation is also found in palmar plantar pustulosis and acrodermatitis continua of Hallopeau. Furthermore, in patients with pre-existing plaque-type psoriasis, the gain of function mutation in *CARD14*, p.Asp176His, was found to be a predisposing factor for developing GPP [120].

In addition to studies of genetic variants, the profiling of gene expression in psoriasis has aided in the understanding of the relevant pathophysiological pathways. Transcriptomic studies of psoriatic skin have revealed differentially expressed genes (DEGs) when compared to healthy skin, and also between lesional and nonlesional psoriatic skin [121,122]. Further underscoring their relevance in psoriasis pathogenesis, IL-17A genes were found to be upregulated in nonlesional psoriatic skin compared to healthy skin. This finding suggests that nonlesional psoriatic skin is also subclinically affected, and supports the concept of the widespread inflammation that is present in psoriasis [123]. In addition, data showing the upregulation of Th2 genes in nonlesional psoriatic skin may reflect the activation of T cell regulatory compensation mechanisms in an effort to override the inflammatory cascade [123]. ‘Cross-disease’ transcriptomics have aided in differentiating nonspecific DEGs present in inflammatory skin conditions (such as atopic dermatitis and squamous cell carcinoma) from DEGs specific to psoriasis. The latter are induced by IL-17A and are expressed by keratinocytes [124].

Despite solid evidence of genetic relevance in the pathogenesis of psoriasis, no single genetic variant seems to be sufficient to account on its own for the development of disease. Hence, a multifactorial setting including multiple genetic mutations and environmental factors, which have been attributed up to 30% of disease risk, ought to be considered [125].

### 2.5. Epigenetics

The quest for the missing heritability associated with psoriasis candidate genes has fueled the search for epigenetic modifications. Epigenetic mechanisms modify gene expression without changing the genomic sequence; some examples include: long noncoding RNA (lncRNA), microRNA (miRNA) silencing, and cytosine and guanine (CpG) methylation.

lncRNA are at least 200 nucleotides long, and are not transcribed to protein. At least 971 lncRNAs have been found to be differentially expressed in psoriatic plaques compared to normal skin [126,127,128,129,130,131]. Thereof, three differentially expressed lncRNAs in proximity to known psoriasis susceptibility loci at *CARD14, LCE3B/LCE3C*, and *IL-23R*, and are thought to modulate their function [127].

miRNAs are small, evolutionarily conserved, noncoding RNAs that base pair with complementary sequences within mRNA molecules, and regulate gene expression at the posttranscriptional level, usually downregulating expression. Most of the studies of miRNAs in association with psoriasis address the plaque-type variant (see Table 1), and so far, more than 250 miRNAs are aberrantly expressed in psoriatic skin [132,133,134,135]. A prominent role has been attributed to miR-31, which is upregulated in psoriatic skin and regulates NF-κB signaling as well as the leukocyte-attracting and endothelial cell-activating signals produced by keratinocytes [135]. miR-21 is an oncomiR with a role in inflammation, and has been found to be elevated in psoriatic skin. Increased miR-21 has been localized not only to the epidermis, but is also found in the dermal inflammatory infiltrates, and correlates with elevated TNF-α mRNA expression [136]. miR-221 and miR-222 are among other upregulated miRNAs in psoriatic skin [132]. The aberrant expression of miR-21, miR-221, and miR-222 correlates with a downregulation of the tissue inhibitor of metalloprotease 3 (TIMP3) [137,138]. TIMP3 is a member of the matrix metalloprotease family with a wide range of functions. Increased levels of said miRs are thought to result in unopposed matrix metalloprotease activity, leading to inflammation (partly via TNF-α-mediated signaling) and epidermal proliferation [138]. miR-210 was found to be highly expressed in psoriasis patients, and induced Th17 and Th1 differentiation while inhibiting Th2 differentiation through STAT6 and LYN repression [139].

Serum levels of miR-33, miR-126, and miR-143, among others, have been proposed as potential biomarkers of disease [140,141]. However, the studies have so far failed to consistently present elevations of a single miRNA in psoriatic patients. Thus, alterations of miRNA expression are better interpreted in the context of miRNA profiles, which have been reported to shift following psoriasis treatments [132]. Thus, miRNA expression profiles could potentially be used to predict response to treatment and personalize therapies.

DNA methylation is another epigenetic mechanism that can alter gene expression in a transient or heritable fashion, and primarily involves the covalent modification of cytosine and guanine (CpG) sequences. CpG methylation is usually repressive unless it inhibits transcriptional repressors, in which case it results in gene activation. Around 1100 differentially methylated CpG sites were detected between psoriatic and control skin. Of these sites, 12 corresponded to genes regulating epidermal differentiation, and were upregulated due to a lower methylation pattern. Said changes in DNA methylation reverted to baseline under anti-TNF-α treatment, indicating that CpG methylation in psoriasis is dynamic [148,149]. Further research will shed light on the functional relevance of epigenetic regulation in psoriasis.

### 2.6. Microbiome

The skin microbiome exerts an active role in immune regulation and pathogen defense by stimulating the production of antibacterial peptides and through biofilm formation. A differential colonizing microbiota in comparison to healthy skin has been found in several dermatologic diseases, including atopic dermatitis, psoriasis, and acne vulgaris [150,151]. It is hypothesized that an aberrant immune activation triggered by skin microbiota is involved in the pathogenesis of autoimmune diseases. For instance, there is growing evidence that the steady-state microbiome plays a role in autoimmune diseases such as in inflammatory bowel disease [152].

The overall microbial diversity is increased in the psoriatic plaque [151]. However, an increase in Firmicutes and Actinobacteria phyla were found in psoriatic plaques (Table 2) [153]. Proteobacteria were found to be higher in healthy skin when compared to psoriatic patients [153,154]. Nevertheless, Proteobacteria were found to be increased in the trunk skin biopsies of psoriatic lesions [151]. A combined increase in Corynebacterium, Propionibacterium, Staphylococcus, and Streptococcus was found in psoriatic skin; however, in another study, Staphylococci were significantly lower in psoriatic skin compared to healthy controls [151,154].

Certain fungi such as Malassezia and Candida albicans, and viruses such as the human papilloma virus have been associated with psoriasis [155]. So far, Malassezia proved to be the most abundant fungus in psoriatic and healthy skin. Nevertheless, the colonization level of Malassezia in psoriasis patients was lower than that in healthy controls [156]. Further studies are required to explain the role of the microbiome signature and the dynamics among different commensal and pathogenic phyla [157]. 

## 3. Therapy

Psoriasis is a chronic relapsing disease, which often necessitates a long-term therapy. The choice of therapy for psoriasis is determined by disease severity, comorbidities, and access to health care. Psoriatic patients are frequently categorized into two groups: mild or moderate to severe psoriasis, depending on the clinical severity of the lesions, the percentage of affected body surface area, and patient quality of life [159]. Clinical disease severity and response to treatment can be graded through a number of different scores. The PASI score has been extensively used in clinical trials, especially those pertaining to the development of the biologic drugs, and will be used throughout this review.

Mild to moderate psoriasis can be treated topically with a combination of glucocorticoids, vitamin D analogues, and phototherapy. Moderate to severe psoriasis often requires systemic treatment. The presence of comorbidities such as psoriasis arthritis is also highly relevant in treatment selection. In this review, we will address the systemic therapies as small-molecule (traditional and new) and biologic drugs.

A number of case reports and case series have suggested that tonsillectomy has a therapeutic effect in patients with guttate psoriasis and plaque psoriasis [69,160,161]. A systematic review concluded that the evidence is insufficient to make general therapeutic recommendations for tonsillectomy, except for selected patients with recalcitrant psoriasis, which is clearly associated to tonsillitis [162]. A recent study stated that HLA-Cw*0602 homozygosity in patients with plaque psoriasis may predict a favorable outcome to tonsillectomy [163]. To date, a single randomized, controlled clinical trial showed that tonsillectomy produced a significant improvement in patients with plaque psoriasis in a two-year follow-up timespan [164]. Furthermore, the same cohort was evaluated to assess the impact of the clinical improvement after tonsillectomy on quality of life. The study reported a 50% improvement in health-related quality of life, and a mean 59% improvement in psoriasis-induced stress. Tonsillectomy was considered worthwhile by 87% of patients who underwent the procedure [165].

### 3.1. Small-Molecule Therapies

In the past years, an accelerated development in psoriasis therapies has resulted in advanced targeted biological drugs. Methotrexate (MTX), cyclosporin A, and retinoids are traditional systemic treatment options for psoriasis. All of the former are oral drugs with the exception of MTX, which is also available for subcutaneous administration. They will be briefly discussed in this review (see Table 3). The section ends with an overview on dimethyl fumarate and apremilast, which are newer drugs that have been approved for psoriasis.

MTX is a folic acid analogue that inhibits DNA synthesis by blocking thymidine and purine biosynthesis. The initial recommended dose of 7.5–10 mg/weekly may be increased to a maximum of 25 mg/weekly [166,167]. A recent retrospective study reported successful treatment response (defined by PASI decrease of 50% to 75% and absolute DLQI value) was reached by 33%, 47%, and 64% of patients at three, six, and 12 months, respectively [168]. There is conflicting evidence regarding MTX effectiveness on psoriatic arthritis. A recent publication reported 22.4% of patients achieved minimal arthritic disease activity, and 27.2% reached a PASI 75 at week 12 [169]. Furthermore, HLA-Cw6 has been suggested as a potential marker for patients who may benefit from MTX treatment [170]. The most common side effects include nausea, leucopenia, and liver transaminase elevation. Despite the potential side effects and its teratotoxicity, it remains a frequently used cost-effective first-line drug, and the close monitoring of liver function and full blood count make a long-term administration feasible.

Cyclosporine is a T cell-inhibiting immunosuppressant from the group of the calcineurin inhibitors. Cyclosporine is effective as a remission inducer in psoriasis and as maintenance therapy for up to two years [171]. Hypertension, renal toxicity, and non-melanoma skin cancer are significant potential side effects. Nephrotoxicity is related to the duration of treatment and the dose. Cyclosporine is employed as an intermittent short-term therapy. The dosage is 2.5 to 5.0 mg/kg of body weight for up to 10 to 16 weeks. Tapering of the drug is recommended to prevent relapse [171].

Retinoids are natural or synthetic vitamin A-related molecules. Acitretin is the retinoid used in the treatment of psoriasis. It affects transcriptional processes by acting through nuclear receptors and normalizes keratinocyte proliferation and differentiation [172,173]. A multicenter, randomized study reported 22.2% and 44.4% of patients reaching PASI 75 and PASI 50 at 24 weeks [174]. Acitretin is initially administered at 0.3–0.5 mg/kg of body weight per day. The maximum dosage is 1 mg/kg body weight/daily. Cheilitis is the most common side effect appearing dose dependently in all patients. Other adverse effects include conjunctivitis, effluvium, hepatitis, and teratogenicity.

Fumaric acid esters (FAEs) are small molecules with immunomodulatory and anti-inflammatory properties [175,176]. The exact mechanism of action has not been cleared, but is thought to involve an interaction with glutathione, which among other mechanisms, inhibits the transcriptional activity of NF-κB [177,178]. FAEs were initially available as a mix of dimethyl fumarate and monoethyl fumarate (DMF/MEF), the former being the main active compound in the formulation. DMF has been reported to decrease the migratory capacity of slan+ monocytes, and also inhibited the induction of Th1/Th17 responses [178]. DMF/MEF was approved in 1994 in Germany for the treatment of severe plaque psoriasis, and in 2008, the indication was expanded for moderate psoriasis [179]. This licensing was exclusive to Germany, where it remains a first-line drug; nevertheless, DMF/MEF was used as off-label treatment in other European countries [180,181,182,183]. A new FAE formulation containing exclusively the main active metabolite DMF became available in 2017, and was approved for psoriasis treatment in the European Union, Iceland, and Norway [184]. Although there are no studies comparing DMF/MEF directly to biologics, several studies document its efficacy [185,186,187,188,189]. A marked improvement is also seen in patients with psoriatic arthritis and nail psoriasis. The most common side effects are gastrointestinal symptoms and flushing, which are generally mild in severity, resolve over time, and are dose related [184]. In addition, FAEs may decrease lymphocyte and leukocyte counts. Therefore, it is recommended to perform a complete blood count before treatment initiation and monthly for DMF/MEF or every three months for DMF [184].

Apremilast, a phosphodiesterase-4 inhibitor, inhibits the hydrolyzation of the second messenger cAMP. This leads to the reduced expression of pro-inflammatory cytokines TNF-α, IFN0γ, and IL-12, and increased levels of IL-10. Apremilast was shown to have broad anti-inflammatory effects on keratinocytes, fibroblasts, and endothelial cells [190]. We studied apremilast in the context of slan^+^ cells, which is a frequent dermal inflammatory dendritic cell type derived from blood circulating slan^+^ nonclassical monocytes. Here, apremilast strongly reduced TNF-α and IL-12 production, but increased IL-23 secretion and IL-17 production in T cells stimulated by apremilast-treated slan^+^ monocytes [191]. These dual effects on slan^+^ antigen-presenting cells may constrain therapeutic responses. No routine monitoring of hematologic parameters is required for apremilast, which is a major advantage compared to the other small molecule drugs. Apremilast showed a 33.1% PASI 75 response at week 16. It is also effective for palmoplantar, scalp psoriasis, and nail psoriasis in addition to psoriatic arthritis [192,193,194]. The most common adverse events affected the gastrointestinal tract (nausea and diarrhea) and the upper respiratory tract (infections and nasopharyngitis). These effects were mild in nature and self-resolving over time.

The traditional systemic drugs are immunomodulators, which except for apremilast require close clinical monitoring due to the common side effects involving mainly the kidney and the liver. Methotrexate and cyclosporine are the only systemic therapies for psoriasis included in the World Health Organization (WHO) Model List of Essential Medicines, albeit for the indications of joint disease for the former and immunosuppression for the latter. The potential side effects of FAE and apremilast are usually not life-threatening, but might be sufficient to warrant discontinuation.

### 3.2. Biologics

In the context of psoriasis treatment, current use of the term *biologics* refers to complex engineered molecules including monoclonal antibodies and receptor fusion proteins. Biologics are different from the above-described systemic therapies in that they target specific inflammatory pathways and are administered subcutaneously (s.c.) (or intravenously i.e., infliximab) on different weekly schedules. Biologics presently target two pathways crucial in the development and chronicity of the psoriatic plaque: the IL-23/Th17 axis and TNF-α-signaling (see Table 3).

#### 3.2.1. TNF-α

TNF-α inhibitors have been available for over a decade. They are considered the first-generation biologics, and are effective for plaque psoriasis and psoriatic arthritis. TNF-α inhibitors are still the standard used to evaluate drug efficacy in psoriasis clinical research. There are currently four drugs in this category: etanercept, infliximab, adalimumab, and certolizumab.

Etanercept is unique in the biologics category in that it is not a monoclonal antibody, but rather a recombinant human fusion protein. The receptor portion for the TNF-α ligand is fused to the Fc portion of an IgG1 antibody. It was the first TNF-α inhibitor approved by the United States Food and Drug Administration (FDA) for psoriasis. Infliximab is a chimeric monoclonal IgG1 antibody, and adalimumab is a fully human monoclonal IgG1 antibody. They neutralize TNF-α activity by binding to its soluble and membrane-bound form. These drugs are particularly employed to treat psoriatic arthritis, and show a similar efficacy. In the treatment of psoriasis, they show different PASI 75 response rates: 52% for etanercept, 59% for adalimumab, and 80% for infliximab. Infliximab shows superiority in terms of efficacy when compared to the other TNF-α inhibitors, and when compared with ustekinumab, it showed a similar performance [195]. The chimeric nature of infliximab might contribute to a higher immunogenic potential of the drug, which in turn might influence drug survival. Certolizumab pegol is a pegylated Fab’ fragment of a humanized monoclonal antibody against TNF-α. PEGylation is the covalent conjugation of proteins with polyethylene glycol (PEG), and is attributed a number of biopharmaceutical improvements, including increased half-life and reduced immunogenicity [196]. The initial indication for treating Crohn’s disease was extended to psoriatic arthritis and recently to plaque psoriasis. Certolizumab has shown an 83% PASI 75 response. Unlike other anti-TNF-α agents, it has no Fc domain, and is thus not actively transported across the placenta. Thus, certolizumab pegol is approved for use during pregnancy and breastfeeding.

#### 3.2.2. IL23/Th17 axis

As previously mentioned, IL-23 drives the expansion of Th17 cells whose inflammatory effects are in turn mediated by IL-17A, IL-17F, and IL-22.

##### IL-23

IL-23 is a dimer composed of p40 and p19. The first biologic to be approved for psoriasis vulgaris after the TNF-α inhibitors was ustekinumab, which is a monoclonal antibody directed against the p40 subunit. P40 is not exclusive to IL-23, but rather is shared with IL-12. IL-12 is a dimer consisting of p40 and p35, and is involved in the differentiation of naïve T cells into Th1 cells. By targeting p40, ustekinumab blocks two different T-cell activating mechanisms, namely Th1 and Th17 selection. Ustekinumab is also effective for the treatment of PsA and Chron’s disease. It is available in two dosages, 45 mg and 90 mg, depending on a threshold body weight of 100 kg. Ustekinumab has extensive safety data, few side effects, good clinical efficacy, and long treatment drug survival was reported. At 90 mg, ustekinumab showed a PASI 75 response in 72.4% and in 61.2% at 45 mg [197]. Studies using real-life data compared ustekinumab with the anti-TNF-α drugs, and ustekinumab was found to have a significant longer drug survival [198,199,200]. Frequent adverse events include nasopharyngitis, upper respiratory tract infections, fatigue, and headache. Among the serious adverse events listed in the label of ustekinumab are infections. Tuberculosis (TB) has only been reported in two psoriasis patients receiving ustekinumab [201,202]. The clinical efficacy of ustekinumab and the further clarification of its mechanism of action highlighted the crucial role of IL-23 in shaping the Th17 response. On the other hand, Th1 signaling is important for the response against bacterial and viral pathogens, and a study showed IL-12 signaling to have a protective effect in a model of imiquimod psoriasis-like inflammation [203]. This rationale fueled the development of drugs targeting p19, which is the IL-23-exclusive subunit. This more specific molecular targeting approach has also achieved successful clinical outcomes. Three fully human monoclonal antibodies with p19 specificity are available: guselkumab, tildrakizumab, and risankizumab. Guselkumab is licensed for psoriasis, and showed clinical superiority when compared to adalimumab, with 85.1% of patients reaching a PASI 75, and 73.3% receiving a PASI 90 response at week 16 [204,205]. Patients receiving tildrakizumab showed a 74% PASI 75, and 52% PASI 90 at week 16. Tildrakizumab was compared to etanercept, and was more likely to reach PASI 75 at weeks 16 and 28 [206,207]. Risankizumab showed the following PASI responses at week 12: 88% PASI 75, 81% PASI 90, and 48% PASI 100. Patients were followed for 48 weeks after the last injection at week 16, and one-fourth of them showed a maintained PASI 100 [208]. Whether IL-23 inhibition has the potential to modify the course of the disease after subsequent drug retrieval is currently under study.

##### IL-17

So far, three human monoclonal antibodies targeting IL-17 are available. Secukinumab and ixekizumab block IL-17A; whereas brodalumab is directed against the IL-17 receptor A. IL-17-targeted biologics are fast acting, showing significant differences from placebo within the first week of treatment. Secukinumab was the first IL-17A inhibitor approved for psoriasis in 2015. A year later, the approval extended to include PsA and ankylosing spondylitis. At week 12, 81.6% of patients on secukinumab reached a PASI 75 response, and 28.6% reached a PASI 100 response [209]. At week 52, over 80% maintained PASI 75. Secukinumab showed a rapid onset of action, reflecting a significant likelihood of achieving PASI 75 as early as the first week of treatment when compared to ustekinumab, and surpassed the latter in clinical superiority at week 16 and 52 [210,211].

Ixekizumab also showed a significantly rapid onset of action in the first week when compared to placebo: a 50% PASI 75 response at week four, and 50% PASI 90 by week eight. At week 12, response rates were 89.1% for PASI 75 and 35.3% for PASI 100 [212]. Secukinumab and ixekizumab have proven effective for scalp and nail psoriasis, which are two clinical variants that are resistant to conventional topical therapies.

Brodalumab is a human monoclonal antibody that targets the IL-17 receptor type A, thus inhibiting the biological activity of IL-17A, IL-17F, interleukin-17A/F, and interleukin-17E (also called interleukin-25). Brodalumab showed an 83.3% PASI 75, 70.3% PASI 90, and 41.9% PASI 100 response rate at week 12, and a satisfactory safety profile [213,214]. After the discontinuation of treatment with secukinumab, 21% of patients maintained their response after one year and 10% after two years [215]. This finding suggests that targeting IL-17 signaling exerts some disease-modifying effect that might reestablish the homeostasis of the inflammatory pathways in a subset of psoriasis patients. Frequent adverse effects under IL-17 blockade include nasopharyngitis, headache, upper respiratory tract infection, and arthralgia. Furthermore, IL-17 signaling is critical for the acute defense against extracellular bacterial and fungal infections. Candida infections are more frequent in patients receiving anti-IL17 biologics secukinumab and ixekizumab compared to etanercept [209]. Nonetheless, candida infections were not severe, and did not warrant treatment interruption. The risk of tuberculosis reactivation is considered small under biologic therapies other than anti-TNF-α [216]. Anti-IL-17 biologics should not be used in psoriasis patients also suffering from Chron’s disease.

#### 3.2.3. Biosimilars in Psoriasis

The introduction of biosimilars for different diseases is revolutionizing the pharmaceutical arsenal at hand. As patents for many biologics face expiration, biosimilar versions of these drugs are being developed, or are already entering the market. A biosimilar is a biological product that must fulfill two requirements: it must be highly similar to an approved biologic product and have no clinically meaningful differences in safety, purity, or potency when compared with the reference product. Guidelines for the development and approval of biosimilars have been issued by the European Medicines Agency, the FDA, and the World Health Organization. There are currently eight adalimumab biosimilars, four infliximab biosimilars, and two etanercept biosimilars approved in Europe. By lowering the costs of systemic treatment for psoriasis patients, biosimilars may also increase access to biologics.

#### 3.2.4. Drugs in the Research Pipeline

Tofacitinib is an oral Janus kinase (JAK) inhibitor currently approved for the treatment of rheumatoid arthritis (RA) and PsA. Tofacitinib showed a 59% PASI 75 and 39% PASI 90 response rate at week 16, and was also effective for nail psoriasis; however, its development for psoriasis was halted for reasons unrelated to safety. Upadacitinib is another JAK inhibitor currently undergoing phase III clinical trials for the treatment of psoriatic arthritis. Piclidenoson, an adenosine A3 receptor inhibitor, serlopitant, a neurokinin-1 receptor antagonist, and RORγt inhibitors are each being tested as oral treatments for psoriasis [217]. Two different biologics targeting IL-17 and one targeting IL-23 are being currently tested. In addition, there are currently 13 registered phase III clinical trials testing biosimilars for adalimumab (eight), infliximab (three), and etanercept (two).

## 4. Outlook

Psoriasis is a complex multifactorial disease for which various novel therapies have arisen in the past years. In spite of the refinement of the targeted therapies, psoriasis remains a treatable but so far not curable disease. The targeted therapies show high clinical efficacy for the inhibition of IL-23 and IL-17. Some degree of a persistent antipsoriatic effect by these therapies could be demonstrated after drug discontinuation, and argue for disease modification concept [208,215]. This important finding will be followed up in ongoing and future studies. However, in other cases, an initial clinical response is only short lived, requiring treatment with a different biologic. Clearly, more research is required to answer the question of why the drug survival of some biologics is limited. The therapeutic arsenal for psoriasis is likely to increase in the near future, with studies on orally applied new small molecules such as inhibitors targeting RORγt. In spite of the safety and efficacy of targeted therapies, due to economic factors, dosage regimes, and adverse effect profiles, broader-acting drugs remain the mainstay of psoriasis systemic therapy in many clinical scenarios around the world. The role of genetics remains to be elucidated not only in the context of predisposition to disease, but also in the profiling of distinct psoriatic types based on cytokine signatures, and in identifying therapy response markers. Clearly, psoriasis is currently the best understood and the best treatable Th17-biased chronic inflammatory disease. After achieving excellent clinical responses for the majority of patients with available therapeutic approaches, the stratification of psoriasis patients to the optimal drug and ensuring the sustainability of our treatments are the major tasks to be resolved.

## Figures and Tables

**Table 1:** MicroRNAs (miRNAs) increased in psoriasis.

miRNA	Target Genes	Tissue/Cell Type (Human)	Function
miR-21	*TIMP3, TPM1, PDCD4, PTEN, IL12A, RECK, RTN4, NFIB*	Skin, PBMCs	Keratinocyte differentiation and proliferation, T cell activation, inflammation [136]
miR-31	*FIH-1, STK40*	Skin	NF-κB activity, keratinocyte differentiation and proliferation [135]
miR-135b	*COL4A3*	Skin	Keratinocyte differentiation and proliferation [137]
miR-146a	*IRAK1, TRAF6, EGFR*	Skin	Hematopoiesis, inflammation, and keratinocyte proliferation [142,143]
miR-155	*CTLA-4*	Skin	Inflammation [144]
miR-203	*TNF-α, IL-8, IL-24, SOCS-3, SOCS-6*	Skin	STAT3 signaling, keratinocyte differentiation and proliferation, and inflammation [145]
miR-210	*FOXP3*	PBMCs	Regulatory T cell activation Induction of Th17 and Th1 differentiation [139,146]
miR-221/222	*TIMP3, c-KIT*	Skin	Immune cell activation Keratinocyte proliferation [138]
miR-424	*MEK1, Cyclin E1*	Skin	Keratinocyte differentiation and proliferation [147]

**Table 2:** Psoriasis microbiome. ↑ increased. > higher than.

Study	Sample (*n*)	Method	Psoriasis	Healthy Skin	Comments
Gao et al., 2008 [153]	Skin swabs (six psoriatic patients)	broad range PCR	↑ diversity ↑ Firmicutes	↑ Actinobacteria ↑ Proteobacteria	Healthy controls taken from previous study [158].
Alekseyenko et al., 2013 [154]	Skin swabs (54 psoriasis patients, 37 controls)	High-throughput 16S rRNA gene sequencing	↑ Actinobacteria/Firmicutes ↑ Corynebacterium, Propionibacterium, Staphylococcus, Streptococcus↑ Corynebacterium, Streptococcus, Staphylococcus	↑ Proteobacteria	OTUs Acidobacteria and Schlegella were strongly associated with psoriasis status. Samples were site-matched.
Fahlen et al., 2012 [151]	Skin biopsies (10 psoriasis patients, 10 healthy controls)	Pyrosequencing targeting the V3-V4 regions of the 16S rRNA gene	Streptococcus > Staphylococcus ↑ Proteobacteria (trunk skin) ↑Propionibacteria/Staph. (limb skin)	↑ Actinobacteria	Included dermis and adnexal structures. Bacterial diversity was increased in the control group (unmatched sites), but not statistically significant. Firmicutes, Proteobacteria, and Actinobacteria predominant in healthy and psoriatic skin.
Takemoto et al., 2015 [156]	Psoriatic scale samples (12 psoriatic patients, 12 healthy controls)	Pyrosequencing for fungal rRNAgene sequences	↑ fungal diversity ↓ Malassezia	↑ Malassezia	Fungal microbiome study Malassezia were the most abundant species in psoriatic and healthy skin.

**Table 3:** Drugs available for psoriasis therapy.

Drug	Mechanism	Application
Methotrexate	Dihydrofolate reductase inhibition blocks purine biosynthesis; induction of lymphocyte apoptosis	s.c./oral
Cyclosporin	Calcineurin inhibition leading to reduced IL-2	Oral
Acitretin	Normalization of keratinocyte proliferation/differentiation through retinoid receptor binding	Oral
Fumarate	Intracellular glutathione, modulation of Nrf2, NF-κB, and HIF-1α; promoting a shift from a pro-inflammatory Th1/Th17 response to an anti-inflammatory/regulatory Th2 response.	Oral
Apremilast	PDE4 inhibitor increases in tracellular cAMP levels in immune and non-immune cell types modulating inflammation	Oral
Etanercept	Dimeric human fusion protein mimicking TNF-αR	s.c.
Infliximab	Chimeric IgG1κ monoclonal antibody that binds to soluble and transmembrane forms of TNF-α	i.v.
Adalimumab	Human monoclonal antibody against TNF-α	s.c.
Certolizumab	Fab portion of humanized monoclonal antibody against TNF-α conjugated to polyethylene glycol	s.c.
Ustekinumab	Human IgG1k monoclonal antibody that binds with specificity to the p40 protein subunit used by both the interleukin (IL)-12 and IL-23 cytokines IL-12/IL-23 p40	s.c.
Tildrakizumab	Humanized IgG1κ, which selectively blocks IL-23 by binding to its p19 subunit	s.c.
Guselkumab	Human immunoglobulin G1 lambda (IgG1λ) monoclonal antibody that selectively blocks IL-23 by binding to its p19 subunit	s.c.
Risankizumab	Humanized IgG1 monoclonal antibody that inhibits interleukin-23 by specifically targeting the p19 subunit	s.c.
Secukinumab	Human IgG1κ monoclonal antibody against IL-17A	s.c.
Ixekizumab	Humanized, immunoglobulin G4κ monoclonal antibody selectively binds and neutralizes IL-17A	s.c.
Brodalumab	Human monoclonal IgG2 antibody directed at the IL-17RA	s.c.

## References

[1] Christophers E. (2001). Psoriasis—Epidemiology and clinical spectrum. Clin. Exp. Dermatol..

[2] Parisi R., Symmons D.P., Griffiths C.E., Ashcroft D.M. (2013). Global epidemiology of psoriasis: A systematic review of incidence and prevalence. J. Investig. Dermatol..

[3] Gibbs S. (1996). Skin disease and socioeconomic conditions in rural Africa: Tanzania. Int. J. Dermatol..

[4] Rachakonda T.D., Schupp C.W., Armstrong A.W. (2014). Psoriasis prevalence among adults in the united states. J. Am. Acad. Dermatol..

[5] Danielsen K., Olsen A.O., Wilsgaard T., Furberg A.S. (2013). Is the prevalence of psoriasis increasing? A 30-year follow-up of a population-based cohort. Br. J. Dermatol..

[6] Ortonne J., Chimenti S., Luger T., Puig L., Reid F., Trueb R.M. (2009). Scalp psoriasis: European consensus on grading and treatment algorithm. J. Eur. Acad. Dermatol. Venereol..

[7] Nestle F.O., Kaplan D.H., Barker J. (2009). Psoriasis. N. Engl. J. Med..

[8] Ko H.C., Jwa S.W., Song M., Kim M.B., Kwon K.S. (2010). Clinical course of guttate psoriasis: Long-term follow-up study. J. Dermatol..

[9] Martin B.A., Chalmers R.J., Telfer N.R. (1996). How great is the risk of further psoriasis following a single episode of acute guttate psoriasis?. Arch. Dermatol..

[10] Navarini A.A., Burden A.D., Capon F., Mrowietz U., Puig L., Koks S., Kingo K., Smith C., Barker J.N., Network E. (2017). European consensus statement on phenotypes of pustular psoriasis. J. Eur. Acad. Dermatol. Venereol..

[11] Sommer D.M., Jenisch S., Suchan M., Christophers E., Weichenthal M. (2006). Increased prevalence of the metabolic syndrome in patients with moderate to severe psoriasis. Arch. Dermatol. Res..

[12] Gerdes S., Mrowietz U., Boehncke W.H. (2016). Comorbidity in psoriasis. Hautarzt.

[13] Ludwig R.J., Herzog C., Rostock A., Ochsendorf F.R., Zollner T.M., Thaci D., Kaufmann R., Vogl T.J., Boehncke W.H. (2007). Psoriasis: A possible risk factor for development of coronary artery calcification. Br. J. Dermatol..

[14] Gelfand J.M., Dommasch E.D., Shin D.B., Azfar R.S., Kurd S.K., Wang X., Troxel A.B. (2009). The risk of stroke in patients with psoriasis. J. Investig. Dermatol..

[15] Prodanovich S., Kirsner R.S., Kravetz J.D., Ma F., Martinez L., Federman D.G. (2009). Association of psoriasis with coronary artery, cerebrovascular, and peripheral vascular diseases and mortality. Arch. Dermatol..

[16] Gelfand J.M., Neimann A.L., Shin D.B., Wang X., Margolis D.J., Troxel A.B. (2006). Risk of myocardial infarction in patients with psoriasis. JAMA.

[17] Ahlehoff O., Gislason G.H., Charlot M., Jorgensen C.H., Lindhardsen J., Olesen J.B., Abildstrom S.Z., Skov L., Torp-Pedersen C., Hansen P.R. (2011). Psoriasis is associated with clinically significant cardiovascular risk: A danish nationwide cohort study. J. Intern. Med..

[18] Kimball A.B., Guerin A., Latremouille-Viau D., Yu A.P., Gupta S., Bao Y., Mulani P. (2010). Coronary heart disease and stroke risk in patients with psoriasis: Retrospective analysis. Am. J. Med..

[19] Stern R.S. (2010). Psoriasis is not a useful independent risk factor for cardiovascular disease. J. Investig. Dermatol..

[20] Stern R.S., Huibregtse A. (2011). Very severe psoriasis is associated with increased noncardiovascular mortality but not with increased cardiovascular risk. J. Investig. Dermatol..

[21] Armstrong E.J., Harskamp C.T., Armstrong A.W. (2013). Psoriasis and major adverse cardiovascular events: A systematic review and meta-analysis of observational studies. J. Am. Heart Assoc..

[22] Gaeta M., Castelvecchio S., Ricci C., Pigatto P., Pellissero G., Cappato R. (2013). Role of psoriasis as independent predictor of cardiovascular disease: A meta-regression analysis. Int. J. Cardiol..

[23] Gu W.J., Weng C.L., Zhao Y.T., Liu Q.H., Yin R.X. (2013). Psoriasis and risk of cardiovascular disease: A meta-analysis of cohort studies. Int. J. Cardiol..

[24] Horreau C., Pouplard C., Brenaut E., Barnetche T., Misery L., Cribier B., Jullien D., Aractingi S., Aubin F., Joly P. (2013). Cardiovascular morbidity and mortality in psoriasis and psoriatic arthritis: A systematic literature review. J. Eur. Acad. Dermatol. Venereol..

[25] Miller I.M., Ellervik C., Yazdanyar S., Jemec G.B. (2013). Meta-analysis of psoriasis, cardiovascular disease, and associated risk factors. J. Am. Acad. Dermatol..

[26] Pietrzak A., Bartosinska J., Chodorowska G., Szepietowski J.C., Paluszkiewicz P., Schwartz R.A. (2013). Cardiovascular aspects of psoriasis: An updated review. Int. J. Dermatol..

[27] Samarasekera E.J., Neilson J.M., Warren R.B., Parnham J., Smith C.H. (2013). Incidence of cardiovascular disease in individuals with psoriasis: A systematic review and meta-analysis. J. Investig. Dermatol..

[28] Xu T., Zhang Y.H. (2012). Association of psoriasis with stroke and myocardial infarction: Meta-analysis of cohort studies. Br. J. Dermatol..

[29] Egeberg A., Skov L., Joshi A.A., Mallbris L., Gislason G.H., Wu J.J., Rodante J., Lerman J.B., Ahlman M.A., Gelfand J.M. (2017). The relationship between duration of psoriasis, vascular inflammation, and cardiovascular events. J. Am. Acad. Dermatol..

[30] Mehta N.N., Yu Y., Saboury B., Foroughi N., Krishnamoorthy P., Raper A., Baer A., Antigua J., Van Voorhees A.S., Torigian D.A. (2011). Systemic and vascular inflammation in patients with moderate to severe psoriasis as measured by [18f]-fluorodeoxyglucose positron emission tomography-computed tomography (FDG-PET/CT): A pilot study. Arch. Dermatol..

[31] Joshi A.A., Lerman J.B., Aberra T.M., Afshar M., Teague H.L., Rodante J.A., Krishnamoorthy P., Ng Q., Aridi T.Z., Salahuddin T. (2016). Glyca is a novel biomarker of inflammation and subclinical cardiovascular disease in psoriasis. Circ. Res..

[32] Ogdie A., Langan S., Love T., Haynes K., Shin D., Seminara N., Mehta N.N., Troxel A., Choi H., Gelfand J.M. (2013). Prevalence and treatment patterns of psoriatic arthritis in the UK. Rheumatology.

[33] Li R., Sun J., Ren L.M., Wang H.Y., Liu W.H., Zhang X.W., Chen S., Mu R., He J., Zhao Y. (2012). Epidemiology of eight common rheumatic diseases in china: A large-scale cross-sectional survey in Beijing. Rheumatology.

[34] Carneiro J.N., Paula A.P., Martins G.A. (2012). Psoriatic arthritis in patients with psoriasis: Evaluation of clinical and epidemiological features in 133 patients followed at the university hospital of Brasilia. An. Bras. Dermatol..

[35] Haroon M., Kirby B., FitzGerald O. (2013). High prevalence of psoriatic arthritis in patients with severe psoriasis with suboptimal performance of screening questionnaires. Ann. Rheum. Dis..

[36] Henes J.C., Ziupa E., Eisfelder M., Adamczyk A., Knaudt B., Jacobs F., Lux J., Schanz S., Fierlbeck G., Spira D. (2014). High prevalence of psoriatic arthritis in dermatological patients with psoriasis: A cross-sectional study. Rheumatol. Int..

[37] Mease P.J., Gladman D.D., Papp K.A., Khraishi M.M., Thaci D., Behrens F., Northington R., Fuiman J., Bananis E., Boggs R. (2013). Prevalence of rheumatologist-diagnosed psoriatic arthritis in patients with psoriasis in European/North American dermatology clinics. J. Am. Acad. Dermatol..

[38] Reich K., Kruger K., Mossner R., Augustin M. (2009). Epidemiology and clinical pattern of psoriatic arthritis in germany: A prospective interdisciplinary epidemiological study of 1511 patients with Plaque-type psoriasis. Br. J. Dermatol..

[39] Villani A.P., Rouzaud M., Sevrain M., Barnetche T., Paul C., Richard M.A., Beylot-Barry M., Misery L., Joly P., Le Maitre M. (2015). Prevalence of undiagnosed psoriatic arthritis among psoriasis patients: Systematic review and meta-analysis. J. Am. Acad. Dermatol..

[40] Stoll M.L., Zurakowski D., Nigrovic L.E., Nichols D.P., Sundel R.P., Nigrovic P.A. (2006). Patients with juvenile psoriatic arthritis comprise two distinct populations. Arthritis Rheum..

[41] Salomon J., Szepietowski J.C., Proniewicz A. (2003). Psoriatic nails: A prospective clinical study. J. Cutan. Med. Surg..

[42] Pasch M.C. (2016). Nail psoriasis: A review of treatment options. Drugs.

[43] Langenbruch A., Radtke M.A., Krensel M., Jacobi A., Reich K., Augustin M. (2014). Nail involvement as a predictor of concomitant psoriatic arthritis in patients with psoriasis. Br. J. Dermatol..

[44] Maejima H., Taniguchi T., Watarai A., Katsuoka K. (2010). Evaluation of nail disease in psoriatic arthritis by using a modified nail psoriasis severity score index. Int. J. Dermatol..

[45] Ellinghaus D., Ellinghaus E., Nair R.P., Stuart P.E., Esko T., Metspalu A., Debrus S., Raelson J.V., Tejasvi T., Belouchi M. (2012). Combined analysis of genome-wide association studies for crohn disease and psoriasis identifies seven shared susceptibility loci. Am. J. Hum. Genet..

[46] Wellcome Trust Case Control Consortium (2007). Genome-wide association study of 14,000 cases of seven common diseases and 3000 shared controls. Nature.

[47] Yeung H., Takeshita J., Mehta N.N., Kimmel S.E., Ogdie A., Margolis D.J., Shin D.B., Attor R., Troxel A.B., Gelfand J.M. (2013). Psoriasis severity and the prevalence of major medical comorbidity: A population-based study. JAMA Dermatol..

[48] Wan J., Wang S., Haynes K., Denburg M.R., Shin D.B., Gelfand J.M. (2013). Risk of moderate to advanced kidney disease in patients with psoriasis: Population based cohort study. BMJ.

[49] Rapp S.R., Feldman S.R., Exum M.L., Fleischer A.B., Reboussin D.M. (1999). Psoriasis causes as much disability as other major medical diseases. J. Am. Acad. Dermatol..

[50] Szepietowski J.C., Reich A. (2016). Pruritus in psoriasis: An update. Eur. J. Pain.

[51] Fleming P., Bai J.W., Pratt M., Sibbald C., Lynde C., Gulliver W.P. (2017). The prevalence of anxiety in patients with psoriasis: A systematic review of observational studies and clinical trials. J. Eur. Acad. Dermatol. Venereol..

[52] Sampogna F., Tabolli S., Abeni D. (2012). Living with psoriasis: Prevalence of shame, anger, worry, and problems in daily activities and social life. Acta Derm. Venereol..

[53] Di Meglio P., Villanova F., Nestle F.O. (2014). Psoriasis. Cold Spring Harb. Perspect. Med..

[54] Harden J.L., Krueger J.G., Bowcock A.M. (2015). The immunogenetics of psoriasis: A comprehensive review. J. Autoimmun..

[55] Liang Y., Sarkar M.K., Tsoi L.C., Gudjonsson J.E. (2017). Psoriasis: A mixed autoimmune and autoinflammatory disease. Curr. Opin. Immunol..

[56] Morizane S., Gallo R.L. (2012). Antimicrobial peptides in the pathogenesis of psoriasis. J. Dermatol..

[57] Morizane S., Yamasaki K., Muhleisen B., Kotol P.F., Murakami M., Aoyama Y., Iwatsuki K., Hata T., Gallo R.L. (2012). Cathelicidin antimicrobial peptide ll-37 in psoriasis enables keratinocyte reactivity against TLR9 ligands. J. Investig. Dermatol..

[58] Nestle F.O., Conrad C., Tun-Kyi A., Homey B., Gombert M., Boyman O., Burg G., Liu Y.J., Gilliet M. (2005). Plasmacytoid predendritic cells initiate psoriasis through interferon-alpha production. J. Exp. Med..

[59] Gregorio J., Meller S., Conrad C., Di Nardo A., Homey B., Lauerma A., Arai N., Gallo R.L., Digiovanni J., Gilliet M. (2010). Plasmacytoid dendritic cells sense skin injury and promote wound healing through type i interferons. J. Exp. Med..

[60] Santini S.M., Lapenta C., Donati S., Spadaro F., Belardelli F., Ferrantini M. (2011). Interferon-α-conditioned human monocytes combine a TH1-orienting attitude with the induction of autologous TH17 responses: Role of IL-23 and IL-12. PLoS ONE.

[61] Hansel A., Gunther C., Ingwersen J., Starke J., Schmitz M., Bachmann M., Meurer M., Rieber E.P., Schakel K. (2011). Human slan (6-sulfo LacNAc) dendritic cells are inflammatory dermal dendritic cells in psoriasis and drive strong TH17/TH1 T-cell responses. J. Allergy Clin. Immunol..

[62] Nestle F.O., Turka L.A., Nickoloff B.J. (1994). Characterization of dermal dendritic cells in psoriasis. Autostimulation of t lymphocytes and induction of th1 type cytokines. J. Clin. Investig..

[63] Van der Fits L., Mourits S., Voerman J.S., Kant M., Boon L., Laman J.D., Cornelissen F., Mus A.M., Florencia E., Prens E.P. (2009). Imiquimod-induced psoriasis-like skin inflammation in mice is mediated via the IL-23/IL-17 axis. J. Immunol..

[64] Matsuzaki G., Umemura M. (2018). Interleukin-17 family cytokines in protective immunity against infections: Role of hematopoietic cell-derived and non-hematopoietic cell-derived interleukin-17s. Microbiol. Immunol..

[65] Gaffen S.L. (2009). Structure and signalling in the IL-17 receptor family. Nat. Rev. Immunol..

[66] Lee J.S., Tato C.M., Joyce-Shaikh B., Gulen M.F., Cayatte C., Chen Y., Blumenschein W.M., Judo M., Ayanoglu G., McClanahan T.K. (2015). Interleukin-23-independent IL-17 production regulates intestinal epithelial permeability. Immunity.

[67] Leung D.Y., Travers J.B., Giorno R., Norris D.A., Skinner R., Aelion J., Kazemi L.V., Kim M.H., Trumble A.E., Kotb M. (1995). Evidence for a streptococcal superantigen-driven process in acute guttate psoriasis. J. Clin. Investig..

[68] Johnston A., Gudjonsson J.E., Sigmundsdottir H., Love T.J., Valdimarsson H. (2004). Peripheral blood t cell responses to keratin peptides that share sequences with streptococcal m proteins are largely restricted to skin-homing CD8^+^ T cells. Clin. Exp. Immunol..

[69] Diluvio L., Vollmer S., Besgen P., Ellwart J.W., Chimenti S., Prinz J.C. (2006). Identical TCR beta-chain rearrangements in streptococcal angina and skin lesions of patients with psoriasis vulgaris. J. Immunol..

[70] Johnston A., Xing X., Wolterink L., Barnes D.H., Yin Z., Reingold L., Kahlenberg J.M., Harms P.W., Gudjonsson J.E. (2017). IL-1 and IL-36 are dominant cytokines in generalized pustular psoriasis. J. Allergy Clin. Immunol..

[71] Bissonnette R., Fuentes-Duculan J., Mashiko S., Li X., Bonifacio K.M., Cueto I., Suarez-Farinas M., Maari C., Bolduc C., Nigen S. (2017). Palmoplantar pustular psoriasis (PPPP) is characterized by activation of the IL-17A pathway. J. Dermatol. Sci..

[72] Wilsmann-Theis D., Schnell L.M., Ralser-Isselstein V., Bieber T., Schon M.P., Huffmeier U., Mossner R. (2018). Successful treatment with interleukin-17a antagonists of generalized pustular psoriasis in patients without IL36RN mutations. J. Dermatol..

[73] Goldminz A.M., Au S.C., Kim N., Gottlieb A.B., Lizzul P.F. (2013). Nf-kappab: An essential transcription factor in psoriasis. J. Dermatol. Sci..

[74] Boutet M.A., Nerviani A., Gallo Afflitto G., Pitzalis C. (2018). Role of the IL-23/IL-17 axis in psoriasis and psoriatic arthritis: The clinical importance of its divergence in skin and joints. Int. J. Mol. Sci..

[75] Sakkas L.I., Bogdanos D.P. (2017). Are psoriasis and psoriatic arthritis the same disease? The IL-23/IL-17 axis data. Autoimmun. Rev..

[76] Mensah K.A., Schwarz E.M., Ritchlin C.T. (2008). Altered bone remodeling in psoriatic arthritis. Curr. Rheumatol. Rep..

[77] Lande R., Botti E., Jandus C., Dojcinovic D., Fanelli G., Conrad C., Chamilos G., Feldmeyer L., Marinari B., Chon S. (2014). The antimicrobial peptide ll37 is a T-cell autoantigen in psoriasis. Nat. Commun..

[78] Arakawa A., Siewert K., Stohr J., Besgen P., Kim S.M., Ruhl G., Nickel J., Vollmer S., Thomas P., Krebs S. (2015). Melanocyte antigen triggers autoimmunity in human psoriasis. J. Exp. Med..

[79] Fuentes-Duculan J., Bonifacio K.M., Hawkes J.E., Kunjravia N., Cueto I., Li X., Gonzalez J., Garcet S., Krueger J.G. (2017). Autoantigens ADAMTSL5 and LL37 are significantly upregulated in active psoriasis and localized with keratinocytes, dendritic cells and other leukocytes. Exp. Dermatol..

[80] Cheung K.L., Jarrett R., Subramaniam S., Salimi M., Gutowska-Owsiak D., Chen Y.L., Hardman C., Xue L., Cerundolo V., Ogg G. (2016). Psoriatic T cells recognize neolipid antigens generated by mast cell phospholipase delivered by exosomes and presented by CD1A. J. Exp. Med..

[81] Yunusbaeva M., Valiev R., Bilalov F., Sultanova Z., Sharipova L., Yunusbayev B. (2018). Psoriasis patients demonstrate HLA-Cw*06:02 allele dosage-dependent T cell proliferation when treated with hair follicle-derived keratin 17 protein. Sci. Rep..

[82] Farber E.M., Nall M.L., Watson W. (1974). Natural history of psoriasis in 61 twin pairs. Arch. Dermatol..

[83] Farber E.M., Nall M.L. (1974). The natural history of psoriasis in 5600 patients. Dermatologica.

[84] Davidson A., Diamond B. (2001). Autoimmune diseases. N. Engl. J. Med..

[85] Hayter S.M., Cook M.C. (2012). Updated assessment of the prevalence, spectrum and case definition of autoimmune disease. Autoimmun. Rev..

[86] Bowcock A.M., Krueger J.G. (2005). Getting under the skin: The immunogenetics of psoriasis. Nat. Rev. Immunol..

[87] Sagoo G.S., Cork M.J., Patel R., Tazi-Ahnini R. (2004). Genome-wide studies of psoriasis susceptibility loci: A review. J. Dermatol. Sci..

[88] Elder J.T. (2018). Expanded genome-wide association study meta-analysis of psoriasis expands the catalog of common psoriasis-associated variants. J. Investig. Dermatol. Symp. Proc..

[89] Trembath R.C., Clough R.L., Rosbotham J.L., Jones A.B., Camp R.D., Frodsham A., Browne J., Barber R., Terwilliger J., Lathrop G.M. (1997). Identification of a major susceptibility locus on chromosome 6p and evidence for further disease loci revealed by a two stage genome-wide search in psoriasis. Hum. Mol. Genet..

[90] Nair R.P., Stuart P.E., Nistor I., Hiremagalore R., Chia N.V., Jenisch S., Weichenthal M., Abecasis G.R., Lim H.W., Christophers E. (2006). Sequence and haplotype analysis supports HLA-C as the psoriasis susceptibility 1 gene. Am. J. Hum. Genet..

[91] Mallon E., Bunce M., Savoie H., Rowe A., Newson R., Gotch F., Bunker C.B. (2000). HLA-C and guttate psoriasis. Br. J. Dermatol..

[92] Gudjonsson J.E., Karason A., Antonsdottir A., Runarsdottir E.H., Hauksson V.B., Upmanyu R., Gulcher J., Stefansson K., Valdimarsson H. (2003). Psoriasis patients who are homozygous for the Hla-Cw*0602 allele have a 2.5-fold increased risk of developing psoriasis compared with Cw6 heterozygotes. Br. J. Dermatol..

[93] Allen M.H., Ameen H., Veal C., Evans J., Ramrakha-Jones V.S., Marsland A.M., Burden A.D., Griffiths C.E., Trembath R.C., Barker J.N. (2005). The major psoriasis susceptibility locus psors1 is not a risk factor for late-onset psoriasis. J. Investig. Dermatol..

[94] Berki D.M., Liu L., Choon S.E., David Burden A., Griffiths C.E.M., Navarini A.A., Tan E.S., Irvine A.D., Ranki A., Ogo T. (2015). Activating card14 mutations are associated with generalized pustular psoriasis but rarely account for familial recurrence in psoriasis vulgaris. J. Investig. Dermatol..

[95] Hwu W.L., Yang C.F., Fann C.S., Chen C.L., Tsai T.F., Chien Y.H., Chiang S.C., Chen C.H., Hung S.I., Wu J.Y. (2005). Mapping of psoriasis to 17q terminus. J. Med. Genet..

[96] Jordan C.T., Cao L., Roberson E.D., Pierson K.C., Yang C.F., Joyce C.E., Ryan C., Duan S., Helms C.A., Liu Y. (2012). PSORS2 is due to mutations in CARD14. Am. J. Hum. Genet..

[97] Tomfohrde J., Silverman A., Barnes R., Fernandez-Vina M.A., Young M., Lory D., Morris L., Wuepper K.D., Stastny P., Menter A. (1994). Gene for familial psoriasis susceptibility mapped to the distal end of human chromosome 17q. Science.

[98] Capon F., Novelli G., Semprini S., Clementi M., Nudo M., Vultaggio P., Mazzanti C., Gobello T., Botta A., Fabrizi G. (1999). Searching for psoriasis susceptibility genes in italy: Genome scan and evidence for a new locus on chromosome 1. J. Investig. Dermatol..

[99] De Cid R., Riveira-Munoz E., Zeeuwen P.L., Robarge J., Liao W., Dannhauser E.N., Giardina E., Stuart P.E., Nair R., Helms C. (2009). Deletion of the late cornified envelope LCE3B and LCE3C genes as a susceptibility factor for psoriasis. Nat. Genet..

[100] Oh I.Y., de Guzman Strong C. (2017). The molecular revolution in cutaneous biology: EDC and locus control. J. Investig. Dermatol..

[101] Riveira-Munoz E., He S.M., Escaramis G., Stuart P.E., Huffmeier U., Lee C., Kirby B., Oka A., Giardina E., Liao W. (2011). Meta-analysis confirms the LCE3C_LCE3B deletion as a risk factor for psoriasis in several ethnic groups and finds interaction with HLA-Cw6. J. Investig. Dermatol..

[102] Elder J.T. (2009). Genome-wide association scan yields new insights into the immunopathogenesis of psoriasis. Genes Immun..

[103] Tsoi L.C., Spain S.L., Ellinghaus E., Stuart P.E., Capon F., Knight J., Tejasvi T., Kang H.M., Allen M.H., Lambert S. (2015). Enhanced meta-analysis and replication studies identify five new psoriasis susceptibility loci. Nat. Commun..

[104] Yin X., Low H.Q., Wang L., Li Y., Ellinghaus E., Han J., Estivill X., Sun L., Zuo X., Shen C. (2015). Genome-wide meta-analysis identifies multiple novel associations and ethnic heterogeneity of psoriasis susceptibility. Nat. Commun..

[105] Tsoi L.C., Spain S.L., Knight J., Ellinghaus E., Stuart P.E., Capon F., Ding J., Li Y., Tejasvi T., Gudjonsson J.E. (2012). Identification of 15 new psoriasis susceptibility loci highlights the role of innate immunity. Nat. Genet..

[106] Parham C., Chirica M., Timans J., Vaisberg E., Travis M., Cheung J., Pflanz S., Zhang R., Singh K.P., Vega F. (2002). A receptor for the heterodimeric cytokine IL-23 is composed of IL-12Rβ1 and a novel cytokine receptor subunit, IL-23R. J. Immunol..

[107] Andres R.M., Hald A., Johansen C., Kragballe K., Iversen L. (2013). Studies of jak/stat3 expression and signalling in psoriasis identifies STAT3-SER727 phosphorylation as a modulator of transcriptional activity. Exp. Dermatol..

[108] Di Meglio P., Di Cesare A., Laggner U., Chu C.C., Napolitano L., Villanova F., Tosi I., Capon F., Trembath R.C., Peris K. (2011). The IL23R R381Q gene variant protects against immune-mediated diseases by impairing IL-23-induced TH17 effector response in humans. PLoS ONE.

[109] Kopp T., Riedl E., Bangert C., Bowman E.P., Greisenegger E., Horowitz A., Kittler H., Blumenschein W.M., McClanahan T.K., Marbury T. (2015). Clinical improvement in psoriasis with specific targeting of interleukin-23. Nature.

[110] Eken A., Singh A.K., Oukka M. (2014). Interleukin 23 in crohn’s disease. Inflamm. Bowel. Dis..

[111] Ghoreschi K., Laurence A., O’Shea J.J. (2009). Selectivity and therapeutic inhibition of kinases: To be or not to be?. Nat. Immunol..

[112] Zhang F., Meng G., Strober W. (2008). Interactions among the transcription factors Runx1, RORγt and Foxp3 regulate the differentiation of interleukin 17-producing T cells. Nat. Immunol..

[113] Craiglow B.G., Boyden L.M., Hu R., Virtanen M., Su J., Rodriguez G., McCarthy C., Luna P., Larralde M., Humphrey S. (2018). CARD14-associated papulosquamous eruption: A spectrum including features of psoriasis and pityriasis rubra pilaris. J. Am. Acad. Dermatol..

[114] Lizzul P.F., Aphale A., Malaviya R., Sun Y., Masud S., Dombrovskiy V., Gottlieb A.B. (2005). Differential expression of phosphorylated NF-κB/RELA in normal and psoriatic epidermis and downregulation of NF-κB in response to treatment with etanercept. J. Investig. Dermatol..

[115] Nair R.P., Duffin K.C., Helms C., Ding J., Stuart P.E., Goldgar D., Gudjonsson J.E., Li Y., Tejasvi T., Feng B.J. (2009). Genome-wide scan reveals association of psoriasis with IL-23 and NF-κB pathways. Nat. Genet..

[116] Stuart P.E., Nair R.P., Ellinghaus E., Ding J., Tejasvi T., Gudjonsson J.E., Li Y., Weidinger S., Eberlein B., Gieger C. (2010). Genome-wide association analysis identifies three psoriasis susceptibility loci. Nat. Genet..

[117] Huffmeier U., Uebe S., Ekici A.B., Bowes J., Giardina E., Korendowych E., Juneblad K., Apel M., McManus R., Ho P. (2010). Common variants at TRAF3IP2 are associated with susceptibility to psoriatic arthritis and psoriasis. Nat. Genet..

[118] Marrakchi S., Guigue P., Renshaw B.R., Puel A., Pei X.Y., Fraitag S., Zribi J., Bal E., Cluzeau C., Chrabieh M. (2011). Interleukin-36-receptor antagonist deficiency and generalized pustular psoriasis. N. Engl. J. Med..

[119] Onoufriadis A., Simpson M.A., Pink A.E., Di Meglio P., Smith C.H., Pullabhatla V., Knight J., Spain S.L., Nestle F.O., Burden A.D. (2011). Mutations in IL36RN/IL1F5 are associated with the severe episodic inflammatory skin disease known as generalized pustular psoriasis. Am. J. Hum. Genet..

[120] Sugiura K. (2014). The genetic background of generalized pustular psoriasis: Il36rn mutations and card14 gain-of-function variants. J. Dermatol. Sci..

[121] Tian S., Krueger J.G., Li K., Jabbari A., Brodmerkel C., Lowes M.A., Suarez-Farinas M. (2012). Meta-analysis derived (mad) transcriptome of psoriasis defines the “core” pathogenesis of disease. PLoS ONE.

[122] Ainali C., Valeyev N., Perera G., Williams A., Gudjonsson J.E., Ouzounis C.A., Nestle F.O., Tsoka S. (2012). Transcriptome classification reveals molecular subtypes in psoriasis. BMC Genom..

[123] Chiricozzi A., Suarez-Farinas M., Fuentes-Duculan J., Cueto I., Li K., Tian S., Brodmerkel C., Krueger J.G. (2016). Increased expression of interleukin-17 pathway genes in nonlesional skin of moderate-to-severe psoriasis vulgaris. Br. J. Dermatol..

[124] Swindell W.R., Stuart P.E., Sarkar M.K., Voorhees J.J., Elder J.T., Johnston A., Gudjonsson J.E. (2014). Cellular dissection of psoriasis for transcriptome analyses and the post-GWAS era. BMC Med. Genom..

[125] Grjibovski A.M., Olsen A.O., Magnus P., Harris J.R. (2007). Psoriasis in norwegian twins: Contribution of genetic and environmental effects. J. Eur. Acad. Dermatol. Venereol..

[126] Gomez J.A., Wapinski O.L., Yang Y.W., Bureau J.F., Gopinath S., Monack D.M., Chang H.Y., Brahic M., Kirkegaard K. (2013). The nest long NCRNA controls microbial susceptibility and epigenetic activation of the interferon-γ locus. Cell.

[127] Gupta R., Ahn R., Lai K., Mullins E., Debbaneh M., Dimon M., Arron S., Liao W. (2016). Landscape of long noncoding RNAS in psoriatic and healthy skin. J. Investig. Dermatol..

[128] Sonkoly E., Bata-Csorgo Z., Pivarcsi A., Polyanka H., Kenderessy-Szabo A., Molnar G., Szentpali K., Bari L., Megyeri K., Mandi Y. (2005). Identification and characterization of a novel, psoriasis susceptibility-related noncoding RNA gene, PRINS. J. Biol. Chem..

[129] Szegedi K., Sonkoly E., Nagy N., Nemeth I.B., Bata-Csorgo Z., Kemeny L., Dobozy A., Szell M. (2010). The anti-apoptotic protein G1P3 is overexpressed in psoriasis and regulated by the non-coding RNA, PRINS. Exp. Dermatol..

[130] Tsoi L.C., Iyer M.K., Stuart P.E., Swindell W.R., Gudjonsson J.E., Tejasvi T., Sarkar M.K., Li B., Ding J., Voorhees J.J. (2015). Analysis of long non-coding RNAS highlights tissue-specific expression patterns and epigenetic profiles in normal and psoriatic skin. Genome Biol..

[131] Wan D.C., Wang K.C. (2014). Long noncoding RNA: Significance and potential in skin biology. Cold Spring Harb. Perspect. Med..

[132] Hawkes J.E., Nguyen G.H., Fujita M., Florell S.R., Callis Duffin K., Krueger G.G., O’Connell R.M. (2016). Micrornas in psoriasis. J. Investig. Dermatol..

[133] Lovendorf M.B., Zibert J.R., Gyldenlove M., Ropke M.A., Skov L. (2014). MicroRNA-223 and MIR-143 are important systemic biomarkers for disease activity in psoriasis. J. Dermatol. Sci..

[134] Paek S.Y., Han L., Weiland M., Lu C.J., McKinnon K., Zhou L., Lim H.W., Elder J.T., Mi Q.S. (2015). Emerging biomarkers in psoriatic arthritis. IUBMB Life.

[135] Xu N., Meisgen F., Butler L.M., Han G., Wang X.J., Soderberg-Naucler C., Stahle M., Pivarcsi A., Sonkoly E. (2013). MicroRNA-31 is overexpressed in psoriasis and modulates inflammatory cytokine and chemokine production in keratinocytes via targeting serine/threonine kinase 40. J. Immunol..

[136] Guinea-Viniegra J., Jiménez M., Schonthaler H.B., Navarro R., Delgado Y., José Concha-Garzón M., Tschachler E., Obad S., Daudén E., Wagner E.F. (2014). Targeting MIR-21 to treat psoriasis. Sci. Transl. Med..

[137] Joyce C.E., Zhou X., Xia J., Ryan C., Thrash B., Menter A., Zhang W., Bowcock A.M. (2011). Deep sequencing of small RNAs from human skin reveals major alterations in the psoriasis miRNAome. Hum. Mol. Genet..

[138] Zibert J.R., Lovendorf M.B., Litman T., Olsen J., Kaczkowski B., Skov L. (2010). Micrornas and potential target interactions in psoriasis. J. Dermatol. Sci..

[139] Wu R., Zeng J., Yuan J., Deng X., Huang Y., Chen L., Zhang P., Feng H., Liu Z., Wang Z. (2018). MicroRNA-210 overexpression promotes psoriasis-like inflammation by inducing TH1 and TH17 cell differentiation. J. Clin. Investig..

[140] Lovendorf M.B., Mitsui H., Zibert J.R., Ropke M.A., Hafner M., Dyring-Andersen B., Bonefeld C.M., Krueger J.G., Skov L. (2015). Laser capture microdissection followed by next-generation sequencing identifies disease-related micrornas in psoriatic skin that reflect systemic microRNA changes in psoriasis. Exp. Dermatol..

[141] Garcia-Rodriguez S., Arias-Santiago S., Orgaz-Molina J., Magro-Checa C., Valenzuela I., Navarro P., Naranjo-Sintes R., Sancho J., Zubiaur M. (2014). Abnormal levels of expression of plasma microRNA-33 in patients with psoriasis. Actas. Dermosifiliogr..

[142] Chatzikyriakidou A., Voulgari P.V., Georgiou I., Drosos A.A. (2010). The role of microrna-146a (miR-146a) and its target IL-1R-associated kinase (IRAK1) in psoriatic arthritis susceptibility. Scand. J. Immunol..

[143] Zhang W., Yi X., Guo S., Shi Q., Wei C., Li X., Gao L., Wang G., Gao T., Wang L. (2014). A single-nucleotide polymorphism of mir-146a and psoriasis: An association and functional study. J. Cell. Mol. Med..

[144] Xu L., Leng H., Shi X., Ji J., Fu J., Leng H. (2017). MiR-155 promotes cell proliferation and inhibits apoptosis by PTEN signaling pathway in the psoriasis. Biomed. Pharmacother..

[145] Primo M.N., Bak R.O., Schibler B., Mikkelsen J.G. (2012). Regulation of pro-inflammatory cytokines TNFα and IL24 by microRNA-203 in primary keratinocytes. Cytokine.

[146] Zhao M., Wang L.T., Liang G.P., Zhang P., Deng X.J., Tang Q., Zhai H.Y., Chang C.C., Su Y.W., Lu Q.J. (2014). Up-regulation of microRNA-210 induces immune dysfunction via targeting FOXP3 in CD4^+^ T cells of psoriasis vulgaris. Clin. Immunol..

[147] Tsuru Y., Jinnin M., Ichihara A., Fujisawa A., Moriya C., Sakai K., Fukushima S., Ihn H. (2014). MiR-424 levels in hair shaft are increased in psoriatic patients. J. Dermatol..

[148] Gudjonsson J.E., Krueger G. (2012). A role for epigenetics in psoriasis: Methylated cytosine-guanine sites differentiate lesional from nonlesional skin and from normal skin. J. Investig. Dermatol..

[149] Roberson E.D., Liu Y., Ryan C., Joyce C.E., Duan S., Cao L., Martin A., Liao W., Menter A., Bowcock A.M. (2012). A subset of methylated CPG sites differentiate psoriatic from normal skin. J. Investig. Dermatol..

[150] Byrd A.L., Belkaid Y., Segre J.A. (2018). The human skin microbiome. Nat. Rev. Microbiol..

[151] Fahlen A., Engstrand L., Baker B.S., Powles A., Fry L. (2012). Comparison of bacterial microbiota in skin biopsies from normal and psoriatic skin. Arch. Dermatol. Res..

[152] Miyoshi J., Chang E.B. (2017). The gut microbiota and inflammatory bowel diseases. Transl. Res..

[153] Gao Z., Tseng C.H., Strober B.E., Pei Z., Blaser M.J. (2008). Substantial alterations of the cutaneous bacterial biota in psoriatic lesions. PLoS ONE.

[154] Alekseyenko A.V., Perez-Perez G.I., De Souza A., Strober B., Gao Z., Bihan M., Li K., Methe B.A., Blaser M.J. (2013). Community differentiation of the cutaneous microbiota in psoriasis. Microbiome.

[155] Fry L., Baker B.S. (2007). Triggering psoriasis: The role of infections and medications. Clin. Dermatol..

[156] Takemoto A., Cho O., Morohoshi Y., Sugita T., Muto M. (2015). Molecular characterization of the skin fungal microbiome in patients with psoriasis. J. Dermatol..

[157] Statnikov A., Alekseyenko A.V., Li Z., Henaff M., Perez-Perez G.I., Blaser M.J., Aliferis C.F. (2013). Microbiomic signatures of psoriasis: Feasibility and methodology comparison. Sci. Rep..

[158] Gao Z., Tseng C.H., Pei Z., Blaser M.J. (2007). Molecular analysis of human forearm superficial skin bacterial biota. Proc. Natl. Acad. Sci. USA.

[159] Mrowietz U., Kragballe K., Reich K., Spuls P., Griffiths C.E., Nast A., Franke J., Antoniou C., Arenberger P., Balieva F. (2011). Definition of treatment goals for moderate to severe psoriasis: A European consensus. Arch. Dermatol. Res..

[160] Hone S.W., Donnelly M.J., Powell F., Blayney A.W. (1996). Clearance of recalcitrant psoriasis after tonsillectomy. Clin. Otolaryngol. Allied Sci..

[161] McMillin B.D., Maddern B.R., Graham W.R. (1999). A role for tonsillectomy in the treatment of psoriasis?. Ear Nose Throat. J..

[162] Rachakonda T.D., Dhillon J.S., Florek A.G., Armstrong A.W. (2015). Effect of tonsillectomy on psoriasis: A systematic review. J. Am. Acad. Dermatol..

[163] Thorleifsdottir R.H., Sigurdardottir S.L., Sigurgeirsson B., Olafsson J.H., Petersen H., Sigurdsson M.I., Gudjonsson J.E., Johnston A., Valdimarsson H. (2016). HLA-Cw6 homozygosity in plaque psoriasis is associated with streptococcal throat infections and pronounced improvement after tonsillectomy: A prospective case series. J. Am. Acad. Dermatol..

[164] Thorleifsdottir R.H., Sigurdardottir S.L., Sigurgeirsson B., Olafsson J.H., Sigurdsson M.I., Petersen H., Arnadottir S., Gudjonsson J.E., Johnston A., Valdimarsson H. (2012). Improvement of psoriasis after tonsillectomy is associated with a decrease in the frequency of circulating T cells that recognize streptococcal determinants and homologous skin determinants. J. Immunol..

[165] Thorleifsdottir R.H., Sigurdardottir S.L., Sigurgeirsson B., Olafsson J.H., Sigurdsson M.I., Petersen H., Gudjonsson J.E., Johnston A., Valdimarsson H. (2017). Patient-reported outcomes and clinical response in patients with moderate-to-severe plaque psoriasis treated with tonsillectomy: A randomized controlled trial. Acta Derm. Venereol..

[166] Revicki D., Willian M.K., Saurat J.H., Papp K.A., Ortonne J.P., Sexton C., Camez A. (2008). Impact of adalimumab treatment on health-related quality of life and other patient-reported outcomes: Results from a 16-week randomized controlled trial in patients with moderate to severe plaque psoriasis. Br. J. Dermatol..

[167] Saurat J.H., Stingl G., Dubertret L., Papp K., Langley R.G., Ortonne J.P., Unnebrink K., Kaul M., Camez A., Investigators C.S. (2008). Efficacy and safety results from the randomized controlled comparative study of adalimumab vs. Methotrexate vs. Placebo in patients with psoriasis (champion). Br. J. Dermatol..

[168] Lindqvist T., Salah L.A., Gillstedt M., Wennberg A.M., Osmancevic A. (2018). Methotrexate management in psoriasis: Are we following the guidelines?. Acta Derm. Venereol..

[169] Coates L.C., Helliwell P.S. (2016). Methotrexate efficacy in the tight control in psoriatic arthritis study. J. Rheumatol..

[170] West J., Ogston S., Berg J., Palmer C., Fleming C., Kumar V., Foerster J. (2017). Hla-cw6-positive patients with psoriasis show improved response to methotrexate treatment. Clin. Exp. Dermatol..

[171] Ho V.C., Griffiths C.E., Berth-Jones J., Papp K.A., Vanaclocha F., Dauden E., Beard A., Puvanarajan L., Paul C. (2001). Intermittent short courses of cyclosporine microemulsion for the long-term management of psoriasis: A 2-year cohort study. J. Am. Acad. Dermatol..

[172] Brand N., Petkovich M., Krust A., Chambon P., de The H., Marchio A., Tiollais P., Dejean A. (1988). Identification of a second human retinoic acid receptor. Nature.

[173] Harper R.A. (1988). Specificity in the synergism between retinoic acid and EGF on the growth of adult human skin fibroblasts. Exp. Cell Res..

[174] Lee J.H., Youn J.I., Kim T.Y., Choi J.H., Park C.J., Choe Y.B., Song H.J., Kim N.I., Kim K.J., Lee J.H. (2016). A multicenter, randomized, open-label pilot trial assessing the efficacy and safety of etanercept 50 mg twice weekly followed by etanercept 25 mg twice weekly, the combination of etanercept 25 mg twice weekly and acitretin, and acitretin alone in patients with moderate to severe psoriasis. BMC Dermatol..

[175] Gesser B., Johansen C., Rasmussen M.K., Funding A.T., Otkjaer K., Kjellerup R.B., Kragballe K., Iversen L. (2007). Dimethylfumarate specifically inhibits the mitogen and stress-activated kinases 1 and 2 (MSK1/2): Possible role for its anti-psoriatic effect. J. Investig. Dermatol..

[176] Lehmann J.C., Listopad J.J., Rentzsch C.U., Igney F.H., von Bonin A., Hennekes H.H., Asadullah K., Docke W.D. (2007). Dimethylfumarate induces immunosuppression via glutathione depletion and subsequent induction of heme oxygenase 1. J. Investig. Dermatol..

[177] Gillard G.O., Collette B., Anderson J., Chao J., Scannevin R.H., Huss D.J., Fontenot J.D. (2015). Dmf, but not other fumarates, inhibits NF-κB activity in vitro in an NRF2-independent manner. J. Neuroimmunol..

[178] Oehrl S., Olaru F., Kunze A., Maas M., Pezer S., Schmitz M., Schakel K. (2017). Controlling the pro-inflammatory function of 6-sulfo LacNAc (slan) dendritic cells with dimethylfumarate. J. Dermatol. Sci..

[179] Reich K., Thaci D., Mrowietz U., Kamps A., Neureither M., Luger T. (2009). Efficacy and safety of fumaric acid esters in the long-term treatment of psoriasis—A retrospective study (future). J. Dtsch. Dermatol. Ges..

[180] Anstey A.V. (2010). Fumaric acid esters in the treatment of psoriasis. Br. J. Dermatol..

[181] Carboni I., De Felice C., De Simoni I., Soda R., Chimenti S. (2004). Fumaric acid esters in the treatment of psoriasis: An italian experience. J. Dermatol. Treat..

[182] Heelan K., Markham T. (2012). Fumaric acid esters as a suitable first-line treatment for severe psoriasis: An irish experience. Clin. Exp. Dermatol..

[183] Kokelj F., Plozzer C., Avian A., Trevisan G. (2009). Fumaric acid and its derivatives in the treatment of psoriasis vulgaris: Our experience in forty-one patients. Acta Dermatovenerol. Croat..

[184] Agency E.M. (2017). Assessment Report: Skilarence.

[185] Altmeyer P.J., Matthes U., Pawlak F., Hoffmann K., Frosch P.J., Ruppert P., Wassilew S.W., Horn T., Kreysel H.W., Lutz G. (1994). Antipsoriatic effect of fumaric acid derivatives. Results of a multicenter double-blind study in 100 patients. J. Am. Acad. Dermatol..

[186] Fallah Arani S., Neumann H., Hop W.C., Thio H.B. (2011). Fumarates vs. Methotrexate in moderate to severe chronic plaque psoriasis: A multicentre prospective randomized controlled clinical trial. Br. J. Dermatol..

[187] Gollnick H., Altmeyer P., Kaufmann R., Ring J., Christophers E., Pavel S., Ziegler J. (2002). Topical calcipotriol plus oral fumaric acid is more effective and faster acting than oral fumaric acid monotherapy in the treatment of severe chronic plaque psoriasis vulgaris. Dermatology.

[188] Nieboer C., de Hoop D., Langendijk P.N., van Loenen A.C., Gubbels J. (1990). Fumaric acid therapy in psoriasis: A double-blind comparison between fumaric acid compound therapy and monotherapy with dimethylfumaric acid ester. Dermatologica.

[189] Nugteren-Huying W.M., van der Schroeff J.G., Hermans J., Suurmond D. (1990). Fumaric acid therapy for psoriasis: A randomized, double-blind, placebo-controlled study. J. Am. Acad. Dermatol..

[190] Schafer P.H., Parton A., Gandhi A.K., Capone L., Adams M., Wu L., Bartlett J.B., Loveland M.A., Gilhar A., Cheung Y.F. (2010). Apremilast, a camp phosphodiesterase-4 inhibitor, demonstrates anti-inflammatory activity in vitro and in a model of psoriasis. Br. J. Pharmacol..

[191] Oehrl S., Prakash H., Ebling A., Trenkler N., Wolbing P., Kunze A., Dobel T., Schmitz M., Enk A., Schakel K. (2017). The phosphodiesterase 4 inhibitor apremilast inhibits th1 but promotes th17 responses induced by 6-sulfo LacNAc (slan) dendritic cells. J. Dermatol. Sci..

[192] Papp K., Reich K., Leonardi C.L., Kircik L., Chimenti S., Langley R.G., Hu C., Stevens R.M., Day R.M., Gordon K.B. (2015). Apremilast, an oral phosphodiesterase 4 (PDE4) inhibitor, in patients with moderate to severe plaque psoriasis: Results of a phase III, randomized, controlled trial (efficacy and safety trial evaluating the effects of apremilast in psoriasis [esteem] 1). J. Am. Acad. Dermatol..

[193] Bissonnette R., Haydey R., Rosoph L.A., Lynde C.W., Bukhalo M., Fowler J.F., Delorme I., Gagne-Henley A., Gooderham M., Poulin Y. (2018). Apremilast for the treatment of moderate-to-severe palmoplantar psoriasis: Results from a double-blind, placebo-controlled, randomized study. J. Eur. Acad. Dermatol. Venereol..

[194] Rich P., Gooderham M., Bachelez H., Goncalves J., Day R.M., Chen R., Crowley J. (2016). Apremilast, an oral phosphodiesterase 4 inhibitor, in patients with difficult-to-treat nail and scalp psoriasis: Results of 2 phase iii randomized, controlled trials (ESTEEM 1 and ESTEEM 2). J. Am. Acad. Dermatol..

[195] Lucka T.C., Pathirana D., Sammain A., Bachmann F., Rosumeck S., Erdmann R., Schmitt J., Orawa H., Rzany B., Nast A. (2012). Efficacy of systemic therapies for moderate-to-severe psoriasis: A systematic review and meta-analysis of long-term treatment. J. Eur. Acad. Dermatol. Venereol..

[196] Pasut G. (2014). Pegylation of biological molecules and potential benefits: Pharmacological properties of certolizumab pegol. BioDrugs.

[197] Kimball A.B., Gordon K.B., Fakharzadeh S., Yeilding N., Szapary P.O., Schenkel B., Guzzo C., Li S., Papp K.A. (2012). Long-term efficacy of ustekinumab in patients with moderate-to-severe psoriasis: Results from the phoenix 1 trial through up to 3 years. Br. J. Dermatol..

[198] Gniadecki R., Bang B., Bryld L.E., Iversen L., Lasthein S., Skov L. (2015). Comparison of long-term drug survival and safety of biologic agents in patients with psoriasis vulgaris. Br. J. Dermatol..

[199] Van den Reek J.M., Zweegers J., Kievit W., Otero M.E., van Lumig P.P., Driessen R.J., Ossenkoppele P.M., Njoo M.D., Mommers J.M., Koetsier M.I. (2014). ‘Happy’ drug survival of adalimumab, etanercept and ustekinumab in psoriasis in daily practice care: Results from the BioCAPTURE network. Br. J. Dermatol..

[200] Warren R.B., Smith C.H., Yiu Z.Z.N., Ashcroft D.M., Barker J., Burden A.D., Lunt M., McElhone K., Ormerod A.D., Owen C.M. (2015). Differential drug survival of biologic therapies for the treatment of psoriasis: A prospective observational cohort study from the British association of dermatologists biologic interventions register (Badbir). J. Investig. Dermatol..

[201] Lynch M., Roche L., Horgan M., Ahmad K., Hackett C., Ramsay B. (2017). Peritoneal tuberculosis in the setting of ustekinumab treatment for psoriasis. JAAD Case Rep..

[202] Tsai T.F., Ho J.C., Song M., Szapary P., Guzzo C., Shen Y.K., Li S., Kim K.J., Kim T.Y., Choi J.H. (2011). Efficacy and safety of ustekinumab for the treatment of moderate-to-severe psoriasis: A phase III, randomized, placebo-controlled trial in Taiwanese and Korean patients (PEARL). J. Dermatol. Sci..

[203] Kulig P., Musiol S., Freiberger S.N., Schreiner B., Gyulveszi G., Russo G., Pantelyushin S., Kishihara K., Alessandrini F., Kundig T. (2016). IL-12 protects from psoriasiform skin inflammation. Nat. Commun..

[204] Blauvelt A., Papp K.A., Griffiths C.E., Randazzo B., Wasfi Y., Shen Y.K., Li S., Kimball A.B. (2017). Efficacy and safety of guselkumab, an anti-interleukin-23 monoclonal antibody, compared with adalimumab for the continuous treatment of patients with moderate to severe psoriasis: Results from the phase iii, double-blinded, placebo- and active comparator-controlled voyage 1 trial. J. Am. Acad. Dermatol..

[205] Gordon K.B., Duffin K.C., Bissonnette R., Prinz J.C., Wasfi Y., Li S., Shen Y.K., Szapary P., Randazzo B., Reich K. (2015). A phase 2 trial of guselkumab versus adalimumab for plaque psoriasis. N. Engl. J. Med..

[206] Reich K., Papp K.A., Blauvelt A., Tyring S.K., Sinclair R., Thaci D., Nograles K., Mehta A., Cichanowitz N., Li Q. (2017). Tildrakizumab versus placebo or etanercept for chronic plaque psoriasis (resurface 1 and resurface 2): Results from two randomised controlled, phase 3 trials. Lancet.

[207] Papp K., Thaci D., Reich K., Riedl E., Langley R.G., Krueger J.G., Gottlieb A.B., Nakagawa H., Bowman E.P., Mehta A. (2015). Tildrakizumab (MK-3222), an anti-interleukin-23p19 monoclonal antibody, improves psoriasis in a phase IIB randomized placebo-controlled trial. Br. J. Dermatol..

[208] Papp K.A., Blauvelt A., Bukhalo M., Gooderham M., Krueger J.G., Lacour J.P., Menter A., Philipp S., Sofen H., Tyring S. (2017). Risankizumab versus ustekinumab for moderate-to-severe plaque psoriasis. N. Engl. J. Med..

[209] Langley R.G., Elewski B.E., Lebwohl M., Reich K., Griffiths C.E., Papp K., Puig L., Nakagawa H., Spelman L., Sigurgeirsson B. (2014). Secukinumab in plaque psoriasis—Results of two phase 3 trials. N. Engl. J. Med..

[210] Thaci D., Blauvelt A., Reich K., Tsai T.F., Vanaclocha F., Kingo K., Ziv M., Pinter A., Hugot S., You R. (2015). Secukinumab is superior to ustekinumab in clearing skin of subjects with moderate to severe plaque psoriasis: Clear, a randomized controlled trial. J. Am. Acad. Dermatol..

[211] Blauvelt A., Reich K., Tsai T.F., Tyring S., Vanaclocha F., Kingo K., Ziv M., Pinter A., Vender R., Hugot S. (2017). Secukinumab is superior to ustekinumab in clearing skin of subjects with moderate-to-severe plaque psoriasis up to 1 year: Results from the clear study. J. Am. Acad. Dermatol..

[212] Gordon K.B., Colombel J.F., Hardin D.S. (2016). Phase 3 trials of ixekizumab in moderate-to-severe plaque psoriasis. N. Engl. J. Med..

[213] Papp K.A., Reich K., Paul C., Blauvelt A., Baran W., Bolduc C., Toth D., Langley R.G., Cather J., Gottlieb A.B. (2016). A prospective phase III, randomized, double-blind, placebo-controlled study of brodalumab in patients with moderate-to-severe plaque psoriasis. Br. J. Dermatol..

[214] Puig L. (2017). Brodalumab: The first anti-IL-17 receptor agent for psoriasis. Drugs Today.

[215] Bagel J., Duffin K.C., Moore A., Ferris L.K., Siu K., Steadman J., Kianifard F., Nyirady J., Lebwohl M. (2017). The effect of secukinumab on moderate-to-severe scalp psoriasis: Results of a 24-week, randomized, double-blind, placebo-controlled phase 3b study. J. Am. Acad. Dermatol..

[216] Cantini F., Nannini C., Niccoli L., Petrone L., Ippolito G., Goletti D. (2017). Risk of tuberculosis reactivation in patients with rheumatoid arthritis, ankylosing spondylitis, and psoriatic arthritis receiving non-anti-TNF-targeted biologics. Mediat. Inflamm..

[217] National Psoriasis Foundation. https://www.Psoriasis.Org/drug-pipeline.

